# Radotinib enhances cytarabine (Ara-C)-induced acute myeloid leukemia cell death

**DOI:** 10.1186/s12885-020-07701-8

**Published:** 2020-12-04

**Authors:** Sook-Kyoung Heo, Eui-Kyu Noh, Ho-Min Yu, Do Kyoung Kim, Hye Jin Seo, Yoo Jin Lee, Jaekyung Cheon, Su Jin Koh, Young Joo Min, Yunsuk Choi, Jae-Cheol Jo

**Affiliations:** 1 Biomedical Research Center, Ulsan University Hospital, University of Ulsan College of Medicine, Ulsan, 44033 Republic of Korea; 2 Department of Hematology and Oncology, Ulsan University Hospital, University of Ulsan College of Medicine, 877 Bangeojinsunhwan-doro, Dong-gu, Ulsan, 44033 Republic of Korea

**Keywords:** Radotinib, Acute myeloid leukemia, Cytarabine, Ara-C, Anti-leukemic activity

## Abstract

**Background:**

Acute myeloid leukemia (AML) is a heterogeneous disease that frequently relapses after standard chemotherapy. Therefore, there is a need for the development of novel chemotherapeutic agents that could treat AML effectively. Radotinib, an oral BCR-ABL tyrosine kinase inhibitor, was developed as a drug for the treatment of chronic myeloid leukemia. Previously, we reported that radotinib exerts increased cytotoxic effects towards AML cells. However, little is known about the effects of combining radotinib with Ara-C, a conventional chemotherapeutic agent for AML, with respect to cell death in AML cells. Therefore, we investigated combination effects of radotinib and Ara-C on AML in this study.

**Methods:**

Synergistic anti-cancer effects of radotinib and Ara-C in AML cells including HL60, HEL92.1.7, THP-1 and bone marrow cells from AML patients have been examined. Diverse cell biological assays such as cell viability assay, Annexin V-positive cells, caspase-3 activity, cell cycle distribution, and related signaling pathway have been performed.

**Results:**

The combination of radotinib and Ara-C was found to induce AML cell apoptosis, which involved the mitochondrial pathway. In brief, combined radotinib and Ara-C significantly induced Annexin V-positive cells, cytosolic cytochrome *C*, and the pro-apoptotic protein Bax in AML cells including HL60, HEL92.1.7, and THP-1. In addition, mitochondrial membrane potential and Bcl-xl protein were markedly decreased by radotinib and Ara-C. Moreover, this combination induced caspase-3 activity. Cleaved caspase-3, 7, and 9 levels were also increased by combined radotinib and Ara-C. Additionally, radotinib and Ara-C co-treatment induced G_0_/G_1_ arrest via the induction of CDKIs such as p21 and p27 and the inhibition of CDK2 and cyclin E. Thus, radotinib/Ara-C induces mitochondrial-dependent apoptosis and G_0_/G_1_ arrest via the regulation of the CDKI–CDK–cyclin cascade in AML cells. In addition, our results showed that combined treatment with radotinib and Ara-C inhibits AML cell growth, including tumor volumes and weights in vivo. Also, the combination of radotinib and Ara-C can sensitize cells to chemotherapeutic agents such as daunorubicin or idarubicin in AML cells.

**Conclusions:**

Therefore, our results can be concluded that radotinib in combination with Ara-C possesses a strong anti-AML activity.

**Supplementary Information:**

The online version contains supplementary material available at 10.1186/s12885-020-07701-8.

## Background

Acute myeloid leukemia (AML) is characterized by the rapid growth of abnormal white blood cells that accumulate in the bone marrow and/or peripheral blood [1, 2]. It is a heterogeneous disease, characterized by numerous cytogenetic and molecular alterations [3]. Therefore, AML is still one of the most difficult malignancies to cure. Moreover, many patients with AML die from disease recurrence and therapy options in both the relapsed and refractory settings of this disease are restricted [4]. In addition, there are unmet needs in AML treatment because the current standard treatment is based on old chemotherapeutic regimens. Therefore, the development of novel chemotherapeutic agents that can treat AML effectively is required.

Cytarabine (1-β-D-arabinofuranosylcytosine, cytosine arabinoside, Ara-C) has been used as a mainstream therapy for AML for over 40 years [5, 6]. It slows down DNA synthesis in the S-phase of the cell cycle, and its action is mainly related to DNA fragmentation and chain termination [7, 8]. Generally, it is prescribed alone or in combination with other drugs. Induction therapy consisting of Ara-C in combination with anthracyclines leads to responses in approximately 60% of adult AML patients [9]. However, the development of resistance and high rates of relapse is a significant obstacle to the successful treatment of AML [10]. Therefore, it is essential to develop potential therapeutic regimens that include new drugs that can maximize the effectiveness of Ara-C.

Radotinib, an oral BCR-ABL tyrosine kinase inhibitor, was developed as a drug for the management of chronic phase-chronic myeloid leukemia (CML-CP) in South Korea [11, 12]. It is an effective inhibitor of naive and kinase-domain mutant BCR-ABL1 [12]. More recently, a phase III clinical trial on the efficacy and safety of radotinib showed the generation of complete cytogenetic responses and major molecular responses in patients newly evaluated with Philadelphia chromosome-positive CML-CP [13]. Radotinib also shows neuroprotective effects in a Parkinson’s disease mouse model [14]. More recently, radotinib was found to activate NK cell cytotoxicity against various Fas-expressing solid cancer cells [15].

We previously demonstrated that radotinib exhibits increased cytotoxicity against diverse AML cells [16]. In addition, it inhibits AML cell proliferation by inducing CDK inhibitors including p21 and p27 [17]. Moreover, radotinib induces apoptosis in differentiated cells from AML blasts [16]. Furthermore, targeting c-KIT (CD117) with radotinib promotes cell death in c-KIT-positive AML [18, 19]. However, little is known about the effects of a combination of radotinib and Ara-C with respect to cell death and cell cycle distribution in AML cells. Here, we show that combination therapy, comprising radotinib and Ara-C, induces AML cell apoptosis, which involves the mitochondrial- and caspase-dependent pathway. Further, we attempt to show that radotinib treatment with Ara-C-based regimens could be clinically evaluated for AML patients.

## Methods

### Reagents

Radotinib was generously gifted by Ilyang Pharmaceutical Co., Ltd., (Seoul, South Korea). Its purity was found to be 99.9% by HPLC analysis [16]. The cell culture plates were obtained from SPL Life Sciences (Pocheon, South Korea). All reagents were obtained from Sigma-Aldrich (St. Louis, MO, USA) unless otherwise indicated. The Apoptosis Detection Kit I was purchased from BD Biosciences (San Jose, CA, USA). The CellTiter 96 AQueous One Solution Cell Proliferation Assay was purchased from Promega (Madison, WI, USA). NE-PER Nuclear and Cytoplasmic Extraction Reagents were obtained from Thermo Scientific (Rockford, IL, USA). All antibodies for western blot were purchased from Cell Signaling Technology (Beverly, MA, USA).

### Patient samples

All patients were newly diagnosed with AML (*n* = 5) at Ulsan University Hospital, Ulsan, South Korea, as described in Supplementary Table 1. Bone marrow samples were collected before administering the first round of chemotherapy.

### Isolation of patient cells and culture

The patient cells were isolated by the density gradient method, as previously described [18]. In brief, bone marrow cells (BMCs) were isolated via density gradient centrifugation at 400×*g* using Lymphoprep (Axis-Shield, Oslo, Norway). They were washed with phosphate-buffered saline (PBS) and cultured in RPMI1640 with 10% FBS and 1% penicillin-streptomycin in a 5% CO_2_ humidified atmosphere at 37 °C.

### Cell culture

The human AML cell lines HL60, HEL92.1.7, and THP-1 in this study were grown as suspension cultures in RPMI-1640 medium with 10% FBS and a 1% penicillin-streptomycin solution (final concentration: 100 units/ml and 100 μg/ml, respectively) in a 5% CO_2_ humidified atmosphere at 37 °C, as previously described [16]. In addition, the human small cell lung cancer (SCLC) cell line H209 were cultured as described previous herein.

### Cell viability assay

The effect of each drug on cell growth both as a single agent and in combination was determined by cell viability assay. Cells were seeded (density, 2 × 10^4^ cells/well) in 96-well plates containing 200 μl medium per well and were incubated with 5 μM radotinib and/or 50 nM Ara-C for 48 h at 37 °C. CellTiter 96 solution (20 μl; Promega, Madison, WI, USA) was added directly to each well, and the plates were incubated for 4 h in a humidified atmosphere of 5% CO_2_ at 37 °C. Absorbance was measured at 490 nm using a SpectraMax iD3 Microplate Reader (Molecular Devices, San Jose, CA, USA). Results are expressed as percent change from baseline conditions determined using four to five culture wells for each experimental condition. The following equation was used: death (% of control) = 100 − cell viability [(OD target group / OD of 0 μM radotinib group) × 100]. In some experiments HL60 cells were treated with various concentrations of radotinib (0, 10, 30, 40 and 50 μM) and Ara-C (0, 40, 80, 120 and 160 nM) for 48 h. Additionally, cells were treated with a combined low dosage of idarubicin and daunorubicin.

### Detection of Annexin V-positive cells

HL60 and HEL92.1.7 cells (1 × 10^5^ cells/ml) were seeded in 24-well plates and treated with 5 μM radotinib and/or 50 nM Ara-C for 48 h at 37 °C. The cells were harvested and washed twice with FACS buffer (PBS containing 0.2% bovine serum albumin and 0.1% NaN_3_). Then, the cells were stained with Annexin V-FITC from the Apoptosis Detection Kit I according to the manufacturer’s instructions. Cells were analyzed using the FACSCalibur flow cytometer and CellQuest Pro software.

### Measurement of caspase-3 activity

Cells were examined using the CaspGLOW™ Fluorescein Active Caspase-3 Staining Kit according to the manufacturer’s instructions (Thermo Fisher Scientific, MA, USA).

### Cell cycle analysis

HL60, HEL92.1.7 and THP-1 cells were treated with 5 μM radotinib and/or 50 nM Ara-C for 48 h at 37 °C. They were then washed twice with PBS and fixed with 70% ethanol overnight at − 20 °C, followed by washing again with PBS and incubation with 0.5 ml PI/RNase stain buffer for 15 min at room temperature. The samples were then analyzed using a FACSCalibur flow cytometer and CellQuest Pro software (BD Biosciences).

### Analysis of mitochondrial membrane potential

HL60 and HEL92.1.7 cells were incubated with 5 μM radotinib and/or 50 nM Ara-C for 48 h at 37 °C, harvested, and washed twice with PBS buffer. Mitochondrial membrane potential (MMP, *ΔΨm*) was evaluated by staining the cells with DiOC_6_(3) for 30 min. After incubation, the cells were harvested and washed. Percentages of DiOC_6_(3)-positive cells were determined using a flow cytometer and CellQuest Pro software.

### Preparation of cytosolic extractions for cytochrome *C* analysis

HEL92.1.7 cells were treated with 5 μM radotinib and/or 50 nM Ara-C for 48 h at 37 °C. Cells were washed with ice-cold PBS, resuspended in cold lysis buffer, and incubated on ice for 30 min. Next, the cytosolic fractions of cells were separated using the NE-PER Nuclear and Cytoplasmic Extraction Reagents according to the manufacturer’s instructions (Thermo Fisher Scientific, MA, USA). The release of cytochrome *C* was analyzed by immunoblotting with an anti-cytochrome *C* mAb.

### Western blotting analysis

Cells were incubated with each drug and their combination for 48 h at 37 °C. They were then washed three times with ice-cold PBS and harvested. Western blotting was performed as previously described [17, 18].

### Xenograft animal model

Specific-pathogen-free five-week-old athymic nude male mice were purchased from Koatech (Pyeongtaek, Korea) and kept in a clean environment of the Ulsan University of Korea (Korea, Ulsan). All mice were housed in standard conditions (12-h light/dark cycle) under constant temperature (22–24 °C) and humidity (50–60%), given free access to food and water, and handled in accordance with the Institutional Animal Care and Use Committee (IACUC) of the University of Ulsan (Ulsan, Korea, Approval No. 0117–07). For anesthesia, mice were injected intraperitoneally with tribromoethanol (250 mg/kg). Mice were sacrificed using carbon dioxide (CO_2_) gas per IACUC protocol.

All mice were naïve to previous experimental manipulations. Each mouse was considered as one experimental unit, and mice were housed in 3–5 mice per cage. To minimize experimental bias, mice were randomized into all prospective treatment cages for in vivo preclinical experiments. The inoculations of tumor cells ex vivo were also blinded. The number of cohorts/mice used in each experiment is described in Supplementary Table 2. The xenograft animal model was generated as previously described [18]. Briefly, HEL92.1.7 tumors were established by subcutaneous injection of 1 × 10^7^ cells into the right flank of five-week-old athymic nude male mice (*n* = 5 per group). To aid precise inoculations, mice were anaesthetized. Once tumors were established, mice were treated with vehicle (0.5% carboxymethylcellulose/DW), 50 mg/kg po radotinib daily, 50 mg/kg ip Ara-C daily every 5/7 day or their combination for up to 24 day. The maximal length and width of the tumor were measured once per week using digital calipers, and the tumor volume (V) was calculated using the following formula: V = (length × width^2^) × 0.5. The mice were sacrificed on days 30–34 following tumor cell implantation. If the size of the subcutaneous tumor exceeded 1000 mm in volume, the animals were excluded from the study and the standard was established for euthanasia before a predetermined time point. All tumors met the criteria, and there were no exclusions. The body weights of the tumor-bearing mice did not change significantly during the duration of study. The tumors were excised and weighed, and each tumor tissue was homogenized for the preparation of cell samples for several analyses including western blotting for specific molecular markers.

### TUNEL assay for measurement of DNA double-strand breaks in tumor tissue

Tumors were frozen in optimal cutting temperature (OCT) compound, and stored at − 80 °C until use. The frozen tissue samples were sectioned by a microtome-cryostat (CM1950, Leica Biosystems, IL, USA). Samples were fixed in 4% paraformaldehyde for 10 min, washed in PBS and then treated with 0.1% Triton X-100 in PBS for 10 min. Then, tumor tissue samples were evaluated for apoptosis using the TUNEL Assay Kit according to the manufacturer’s instructions (Abcam, Cambridge, United Kingdom). Cells were analyzed with a Fluorescence microscope (Olympus, NY, USA).

### Staining of proliferating cell nuclear antigen (PCNA) positive cells in tumor tissue

Under the above experimental conditions, tumor tissue was stained with anti-PCNA monoclonal antibody (mAb) or isotype control mAb at 4 °C for 30 min. The samples were then analyzed with a Fluorescence microscope (Olympus, NY, USA).

### Statistical analysis

Data are presented as means ± the standard error of the mean (SEM) based on at least three independent experiments. All values were evaluated by a one-way analysis of variance followed by Tukey post-hoc test, as implemented by GraphPad Prism 7.0 (GraphPad Software, Inc., La Jolla, USA). Differences were considered significant when *P* < 0.05.

## Results

### Radotinib enhances Ara-C-induced AML cell death in AML cell lines and primary patient samples

**Fig. 1:**
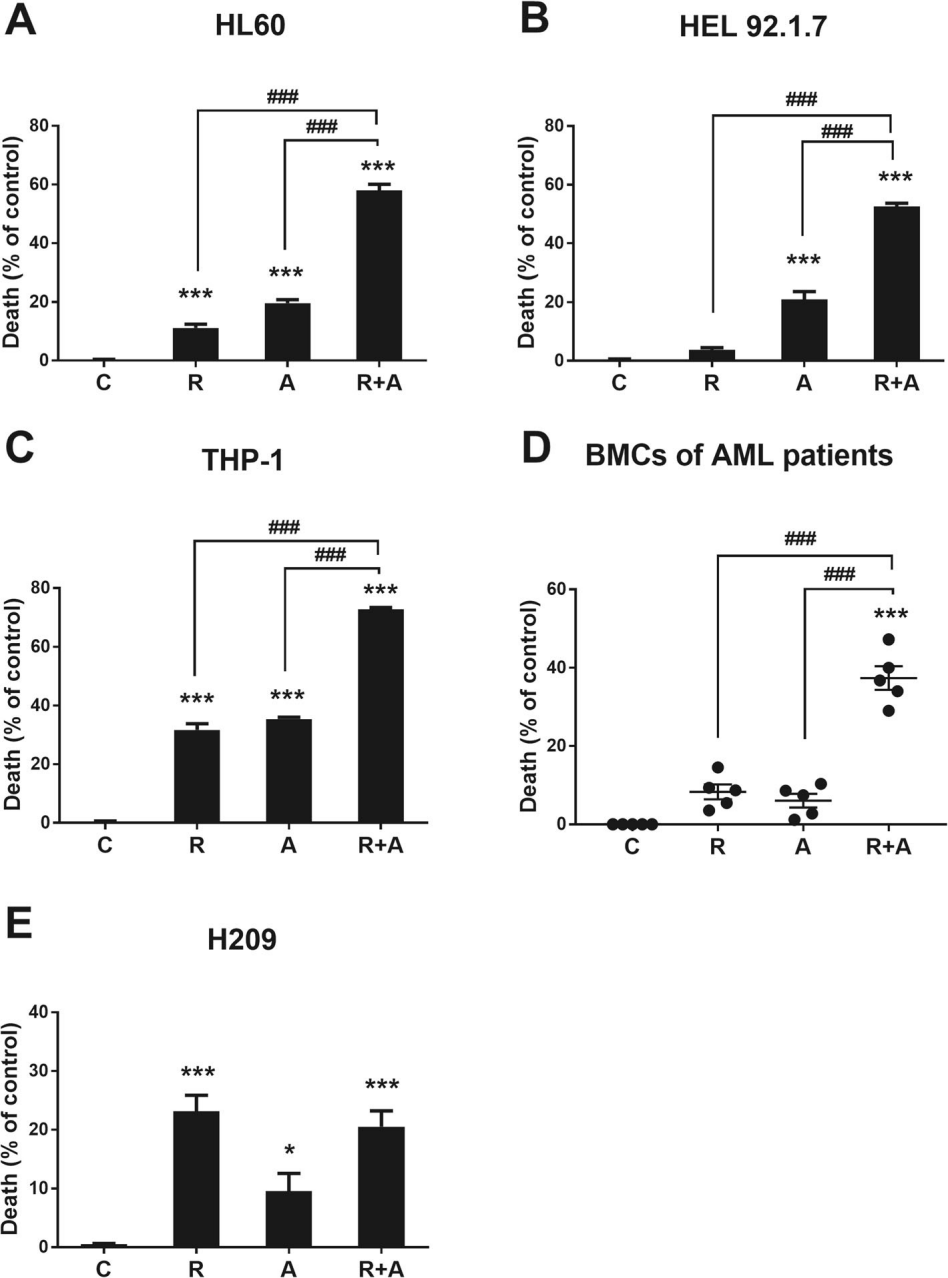
Combination of radotinib and Ara-C enhances acute myeloid leukemia (AML) cell death. Cells were treated with 5 μM radotinib and/or 50 nM Ara-C for 48 h. The cytotoxicity was then evaluated by an MTS assay. **a** HL60 cells. **b** HEL92.1.7 cells. **c** THP-1 cells. **d** Bone marrow cells (BMCs) from AML patients (*n* = 5). **e** H209 cells. Representative data are shown from at least three independent experiments. These data represent the means ± SEM. Significantly different from control (*) or combination of radotinib and Ara-C (#); ***, ###: *P* < 0.001. R, radotinib; A, Ara-C; R + A, combination of radotinib and Ara-C

HL60 cells were treated with various concentrations of radotinib (0, 10, 30, 40 and 50 μM) and Ara-C (0, 40, 80, 120 and 160 nM) for 48 h. Radotinib and Ara-C significantly inhibited the viability of the HL60 cells in a dose-dependent manner (Supplementary Figure 1A and 1B). Interestingly, however, although 5 μM of radotinib and 50 nM of Ara-C alone had little effect on the viability of these cells (over 89 and 81% cell viability, respectively), in combination these concentrations of radotinib and Ara-C produced a significant inhibitory effect of cell viability at 48 h and 72 h (42% at 48 h, 36% at 72 h; see Supplementary Figure 1C and Fig. 1a). Combination effect was better at 48 h than 72 h. Accordingly, we used these conditions for the remainder of the experiments.

We examined the cell viability of diverse AML cells including HL60, HEL 92.1.7, THP-1, and BMCs from AML patients (*n* = 5). Combined treatment with radotinib and Ara-C exerted synergistic effects on AML cell death (Fig. 1a-d). In addition, the human SCLC cell line H209 were tested for cell viability after radotinib and Ara-C treatment. Here, these agents did not have a synergistic effect on cell viability, as expected (Fig. 1e). Therefore, radotinib enhances Ara-C-induced AML, but not MM and SCLC, cell death (Fig. 1).

### Combined treatment with radotinib and Ara-C has synergistic effects on HL60 and HEL92.1.7 cell apoptosis via activation of the mitochondrial- and caspase-dependent apoptosis pathway

**Fig. 2:**
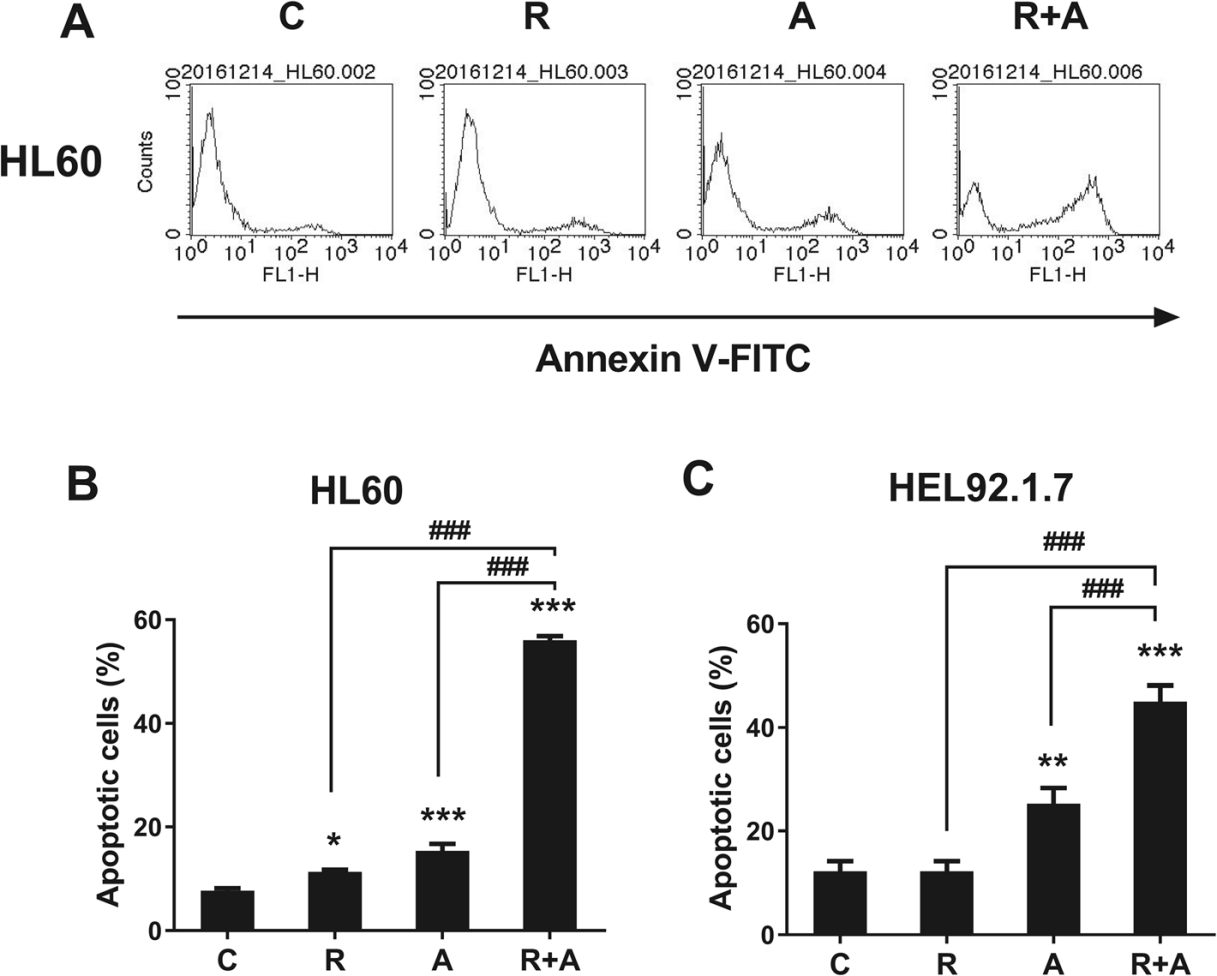
Combination of radotinib and Ara-C increases Annexin V-positive HL60 and HEL92.1.7 cells. Cells were treated with 5 μM radotinib and/or 50 nM Ara-C for 48 h. Cells were stained with annexin V-FITC followed by flow cytometric analysis. **a** Annexin V staining of HL60 cells. **b** Data show the percentage of Annexin V-positive cells (apoptotic cells) in (**a**). **c** Data show the percentage of Annexin V-positive HEL92.1.7 cells. Representative data are shown for at least three independent experiments. These data represent the means ± SEM. Significantly different from control (*) or combination of radotinib and Ara-C (#); *: *P* < 0.05; **: *P* < 0.01; ***, ###: *P* < 0.001. R, radotinib; A, Ara-C; R + A, combination of radotinib and Ara-C

We first observed that combined treatment with radotinib and Ara-C had a synergistic effect on AML cell death. We then performed Annexin V staining to see what pathways regulate this cell death using HL60 and HEL 92.1.7 cells. As a result, we showed that combined treatment with 5 μM radotinib and 50 nM Ara-C exerts a synergistic effect on HL60 and HEL92.1.7 cell apoptosis (Fig. 2a-c).

**Fig. 3:**
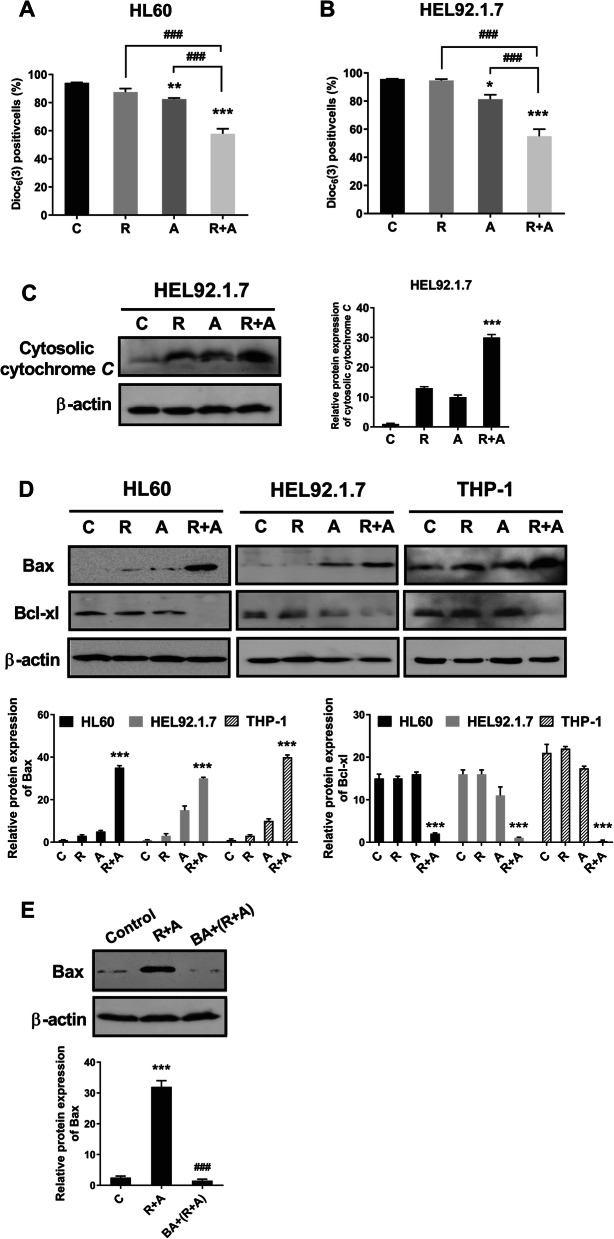
Radotinib/Ara-C-induced apoptosis involves the mitochondrial pathway. A combination of radotinib and Ara-C inhibited the mitochondrial membrane potential of HL60 (**a**) and HEL92.1.7 cells (**b**). The cells were also collected and treated under the same conditions described in Fig. 2. The mitochondrial membrane potential was measured by flow cytometry using DiOC_6_(3) dye. **c** The effects of radotinib/Ara-C on cytosolic cytochrome *C*. HEL92.1.7 cells were incubated with 5 μM radotinib and/or 50 nM Ara-C for 48 h. The cytosolic fractions were then separated, after which each sample was analyzed for the expression of cytochrome *C*. The membrane was stripped and re-probed with an anti-β-actin mAb to confirm equal loading. **d** The effects of radotinib/Ara-C on Bax and Bcl-xl expression. Diverse AML cells including HL60, HEL92.1.7, and THP-1 cells were treated with 5 μM radotinib and/or 50 nM Ara-C for 48 h at 37 °C. Then, cells were washed twice PBS buffer and stained with an anti-Bax and anti-Bcl-xl mAb. **e** HL60 cells were incubated with 5 μM radotinib and 50 nM Ara-C in the presence or absence of bongkrekic acid (BA, 50 μM) for 48 h and then harvested. Cells were analyzed by immunoblotting using an anti-Bax mAb. The membrane was stripped and re-probed with an anti-β-actin mAb to confirm equal loading. These data represent the means ± SEM. Significantly different from control (*) or combination of radotinib and Ara-C (#); ***, ###: *P* < 0.001. R, radotinib; A, Ara-C; R + A, combination of radotinib and Ara-C; BA, bongkrekic acid

Next, we measured the effects of radotinib and Ara-C on the mitochondrial-dependent apoptotic pathway. Cells were collected and MMP was measured by flow cytometry using DiOC_6_(3) dye. As shown in Fig. 3a and b, the DiOC_6_(3)-positive cells were markedly decreased by combined treatment with radotinib and Ara-C stimulation in AML cells including HL60 and HEL92.1.7 cells. Further, as shown in Fig. 3c, cytosolic cytochrome *C* was drastically increased in radotinib and Ara-C-treated HEL92.1.7 cells. In addition, combined radotinib and Ara-C also increased the pro-apoptotic protein Bax in AML cells such as HL60, HEL92.1.7, and THP-1. As expected, this treatment also decreased Bcl-xl expression in diverse AML cells (Fig. 3d). To inhibit MMP disruption, bongkrekic acid (BA, 50 μM) was added to HL60 cells for 2 h prior to combined treatment of radotinib and Ara-C. After 48 h, the cells were analyzed by immunoblotting using an anti-Bax mAb. The BA efficiently blocked the radotinib/Ara-C-induced Bax expression in HL60 cells (relative band density: with 5 μM radotinib and 50 nM Ara-C, 100%; with 50 μM BA preinculation, 4.6%), as shown in Fig. 3e. Thus, these data indicate that radotinib/Ara-C-induces cell death via a mitochondrial-dependent apoptosis pathway (Fig. 3).

**Fig. 4:**
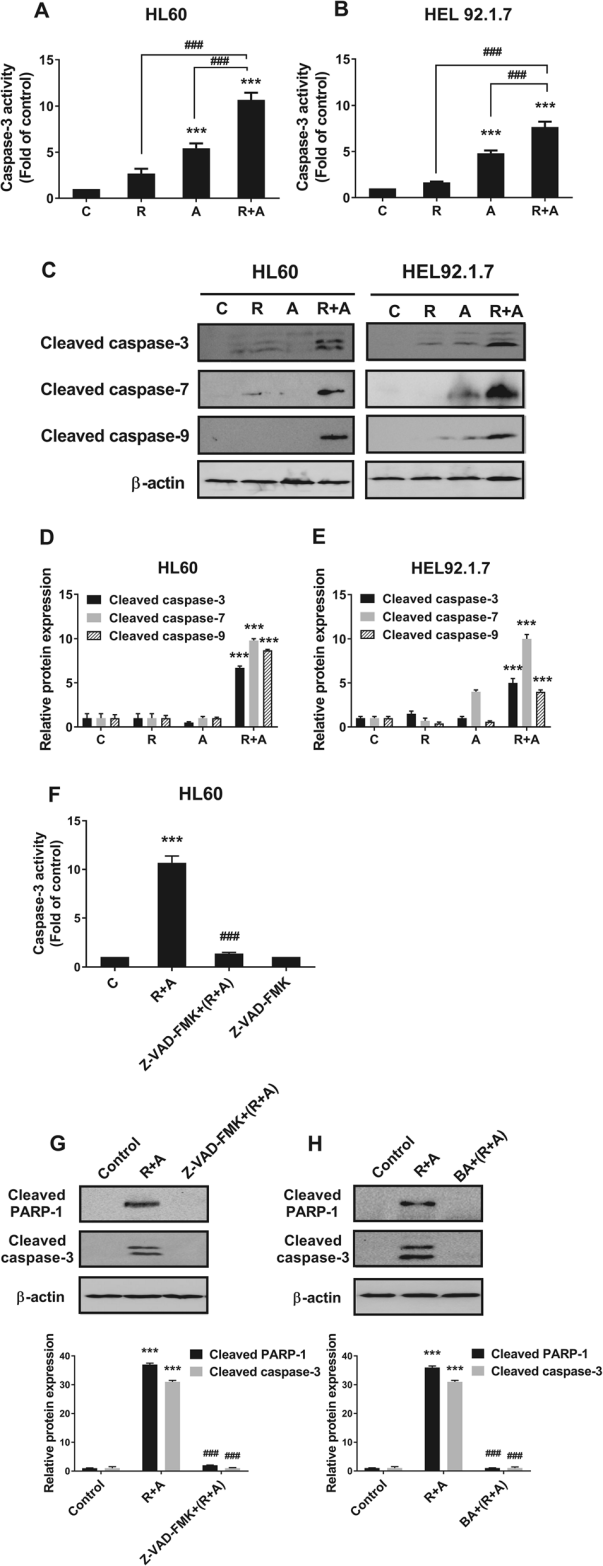
Radotinib/Ara-C induces caspase-dependent apoptosis and activates caspase-3 activity in HL60 and HEL92.1.7 cells. The cells were also collected and treated under the same conditions described in Fig. 2. **a** Caspase-3 activity induced by radotinib and Ara-C stimulation in HL60 cells. **b** Caspase-3 activity induced by radotinib and Ara-C stimulation in HEL92.1.7 cells. **c**-**e** The expression of cleaved caspase-3, cleaved caspase-7, and cleaved caspase-9 was analyzed by SDS-PAGE, followed by western blotting using specific antibodies. **f** HL60 cells were incubated with 5 μM radotinib and 50 nM Ara-C in the presence or absence of Z-VAD-FMK (10 μM) for 48 h and then harvested. Cells were analyzed by capase-3 activity. **g** HL60 cells were also collected and treated under the same conditions described in Fig. 4f. Cells were analyzed by immunoblotting using an anti-cPARP-1 mAb and an anti-cleaved caspase-3 mAb. **h** HL60 cells were also collected and treated under the same conditions described in Fig. 3e. Cells were analyzed by immunoblotting using an anti-cPARP-1 mAb and an anti-cleaved caspase-3 mAb. The experiments were repeated three times and the data show representative results. The membrane was stripped and re-probed with an anti-β-actin mAb to confirm equal loading. These data represent the means ± SEM. Significantly different from control (*) or combination of radotinib and Ara-C (#); ***, ###: *P* < 0.001. R, radotinib; A, Ara-C; R + A, combination of radotinib and Ara-C; BA, bongkrekic acid

We further confirmed that caspase-3 activity was enhanced by treatment with radotinib and Ara-C in HL60 and HEL92.1.7 cells (Fig. 4a and b). At the same time, we observed that cleaved caspase-3, 7, and 9 increased with radotinib/Ara-C administration in both cell types (Fig. 4c-e). To inhibit caspase activation, pan caspase inhibitor, Z-VAD-FMK (10 μM) was added to HL60 cells for 30 min prior to combined treatment of radotinib and Ara-C. After 48 h, the cells were analyzed by capase-3 activity using the CaspGLOW™ Fluorescein Active Caspase-3 Staining Kit. The Z-VAD-FMK efficiently blocked the radotinib/Ara-C-induced capase-3 activity in HL60 cells (relative intensity: with 5 μM radotinib and 50 nM Ara-C, 100%; with 10 μM Z-VAD-FMK preinculation, 4%), as shown in Fig. 4f. The cells were also collected and treated under the same conditions described in Fig. 4f. The cells were analyzed by immunoblotting using an anti-cleaved PARP-1 (cPARP-1) mAb and an anti-cleaved caspase-3 mAb. The Z-VAD-FMK efficiently reversed the radotinib/Ara-C-induced cPARP-1 and cleaved caspase-3 expression in HL60 cells (relative band density of cPARP-1: with 5 μM radotinib and 50 nM Ara-C, 100%; with 10 μM Z-VAD-FMK preinculation, 6%, and relative band density of cleaved caspase-3: with 5 μM radotinib and 50 nM Ara-C, 100%; with 10 μM Z-VAD-FMK preinculation, 2.7%), as shown in as shown in Fig. 4g. Therefore, these data indicate that radotinib/Ara C induces cell death via a caspase-dependent pathway. In addition, the bongkrekic acid powerfully blocked the radotinib/Ara-C-induced cPARP-1 and cleaved caspase-3 expression in HL60 cells (relative band density of cPARP-1: with 5 μM radotinib and 50 nM Ara-C, 100%; with 50 μM BA preinculation, 3.7%, and relative band density of cleaved caspase-3: with 5 μM radotinib and 50 nM Ara-C, 100%; with 50 μM BA preinculation, 3.2%), as shown in as shown in Fig. 4h. Therefore, these data indicate that radotinib/Ara C induces cell death via a mitochondrial-dependent pathway. Generally, it is well-known that the caspase-dependent apoptosis pathway is downstream of the mitochondrial pathway [20]. Therefore, combined treatment with radotinib and Ara-C has a synergistic effect on AML cell death via the activation of mitochondrial-dependent apoptosis.

### Combined treatment with radotinib and Ara-C has a synergistic effect on G_0_/G_1_ phase arrest in HL60 and HEL92.1.7 cells via the induction of p21 and p27

**Fig. 5:**
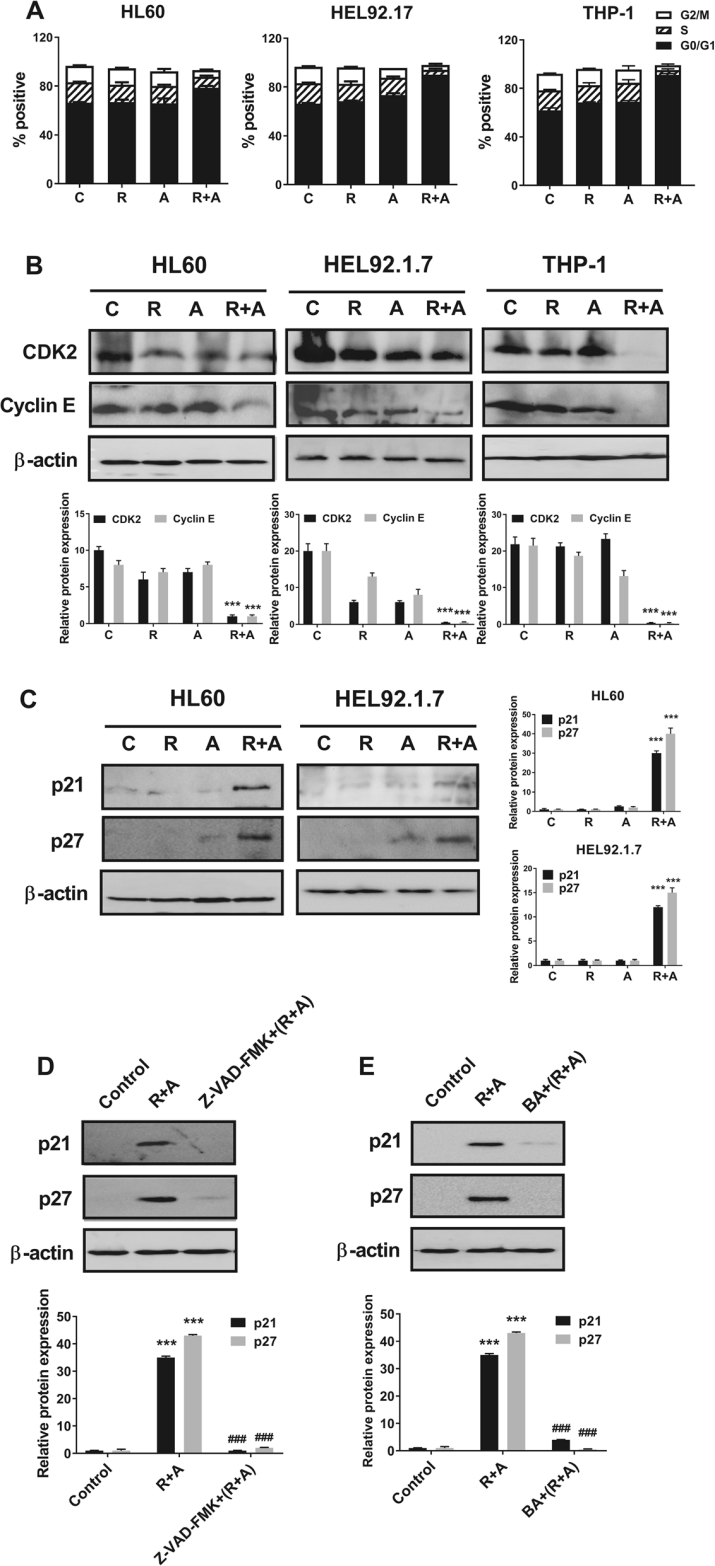
Radotinib/Ara-C induces G_0_/G_1_ phase cell cycle arrest by regulating the CDKI–CDK–cyclin cascade in HL60, HEL92.1.7 and THP-1 cells. **a** Cell cycle distribution at 48 h after radotinib/Ara-C treatment. **b** The expression of CDK2 and cyclin E after radotinib/Ara-C treatment. **c** The expression of CDK inhibitors such as p21 and p27 by western blot analysis following radotinib/Ara-C treatment. **d**, **e** HL60 cells were also collected and treated under the same conditions described in Fig. 4f and g. Cells were analyzed by immunoblotting using an anti-p21 mAb and an anti-p27 mAb. The experiments were repeated three times and the data show representative results. β-Actin was used to confirm equal loading. The results are representative of three independent experiments. R, radotinib; A, Ara-C; R + A, combination of radotinib and Ara-C; BA, bongkrekic acid

We also monitored the cell cycle distribution after radotinib and Ara-C treatment in HL60, HEL92.1.7 and THP-1 cells. First, combination treatment with radotinib and Ara-C caused G_0_/G_1_ phase cell cycle arrest in AML cells such as HL60 and HEL92.1.7 cells (Fig. 5a). Moreover, we examined the effects of combined radotinib and Ara-C on cell cycle regulatory proteins including not only CDK2 and cyclin E but also p21 and p27. As a result, the expression of CDK2 and cyclin E was remarkably decreased in the radotinib and Ara-C combination group (Fig. 5b). Moreover, as shown in Fig. 5c, the expression levels of p21 and p27 in cells co-treated with radotinib and Ara-C were higher than those in single treatment group, as expected. These results suggest that combined treatment with radotinib and Ara-C increases expression of the inhibitory proteins p21 and p27 and decreases the expression of CDK2 and cyclin E in both cell types, maintaining those cells in the G_0_/G_1_ phase. Therefore, the combination of radotinib and Ara-C induces G_0_/G_1_ arrest in HL60 and HEL92.1.7 AML cells via regulation of the CDKI–CDK–cyclin cascade (Fig. 5). Especially, the induction of CDK inhibitors, namely p21 and p27, contributed to the G_0_/G_1_ arrest of HL60 and HEL92.1.7 cells. Consequently, these results indicate that the combination of radotinib and Ara-C has a synergistic effect on G_0_/G_1_-phase arrest in HL60 and HEL92.1.7 cells via the induction of CDK inhibitors including p21 and p27.

Furthermore, we examined cell cycle markers including p21 and p27 using pan caspase inhibitor, Z-VAD-FMK and MMP disruption inhibitor, and bongkrekic acid to clarify the relationship between cell cycle and cell death. In briefly, the cells were also collected and treated under the same conditions described in Fig. 4g and h. The cells were analyzed by immunoblotting using an anti-p21 mAb and an anti-p27 mAb. The Z-VAD-FMK significantly blocked the radotinib/Ara-C-induced p21 and p27 expression in HL60 cells (relative band density of p21: with 5 μM radotinib and 50 nM Ara-C, 100%; with 10 μM Z-VAD-FMK preinculation, 2.6%, and relative band density of p27: with 5 μM radotinib and 50 nM Ara-C, 100%; with 10 μM Z-VAD-FMK preinculation, 4.7%), as shown in as shown in Fig. 5d. In addition, the bongkrekic acid strongly blocked the radotinib/Ara-C-induced (relative band density of p21: with 5 μM radotinib and 50 nM Ara-C, 100%; with 50 μM BA preinculation, 11.4%, and relative band density of p27: with 5 μM radotinib and 50 nM Ara-C, 100%; with 50 μM BA preinculation, 1.2%), as shown in as shown in Fig. 5e. Thus, these data showed that radotinib/Ara C-induced cell cycle markers, p21 and p27, were closely related to AML cell death. It also indicates that mitochondrial-dependent apoptosis and G_0_/G_1_ phase cell cycle arrest contribute to radotinib/Ara-C mediated anti-AML activity.

### Combined treatment with radotinib and Ara-C inhibits AML cell growth in vivo

**Fig. 6:**
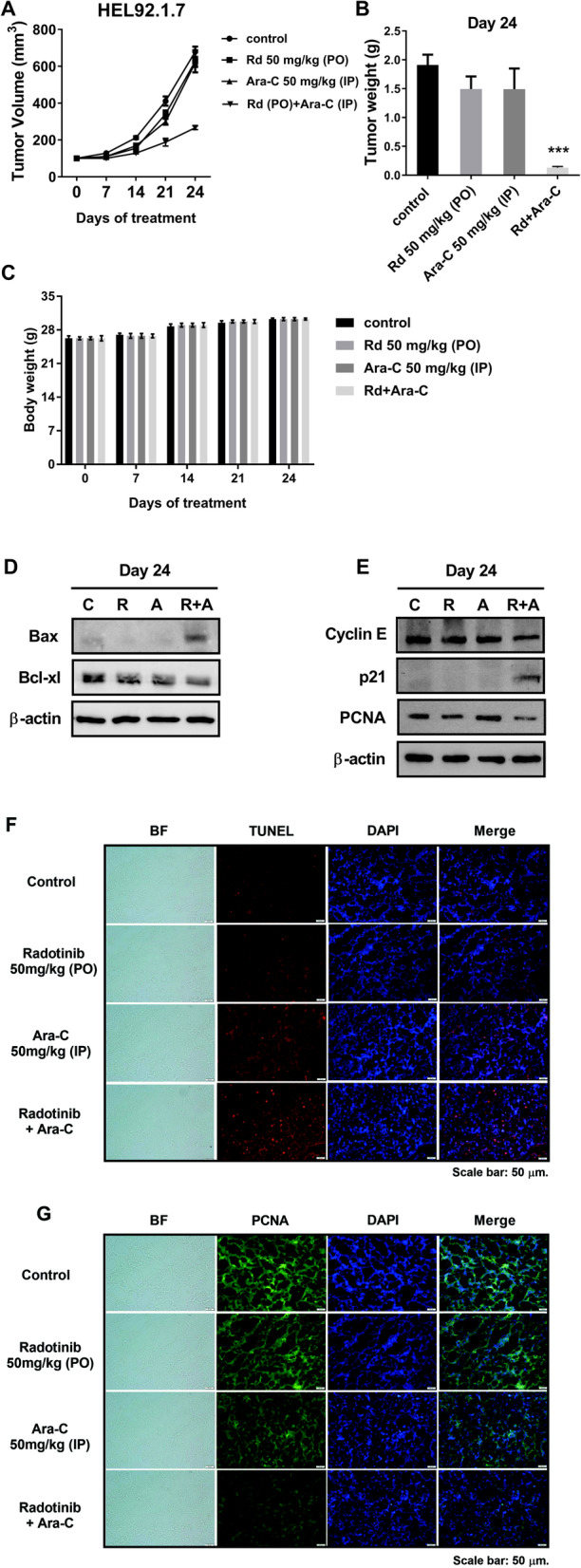
Radotinib and Ara-C inhibit tumor growth in a xenograft animal model using HEL92.1.7 cells. **a** Tumor volume (mm^3^) after radotinib and Ara-C treatment (*n* = 5 for each group). When the tumors were ~ 150 mm^3^ in size at ~ 7 days post-implantation, 0.2 ml radotinib (50 mg/kg body weight, PO) and Ara-C (50 mg/kg body weight, IP) were injected orally and intraperitoneally five times per week. Tumor sizes were measured once per week using digital calipers, and tumor volumes were calculated using the formula (length × width^2^) × 0.5. **b** Tumor weight (g) on day 24. **c** The body weight of mice. **d**, **e** The expression of diverse proteins including Bcl-xl, Cyclin E, PCNA, Bax, and p21 in the tumor. **f** The expression of TUNEL-positive cells in the tumor tissue. **g** The expression of PCNA-positive cells in the tumor tissue. These data represent the means ± SEM. Significantly different from the control (*); ***, *P* < 0.001

To examine the effect of combined treatment with radotinib and Ara-C on AML cell death in vivo, we implanted HEL92.1.7 cells into nude mice. Through previous preliminary studies, radotinib and Ara C were administered by concentration, and then the tumor volume was used in combination with each concentration that decreased by 5–8% compared to the control group (data not shown). As shown in Fig. 6a-b, combined treatment with radotinib and Ara-C inhibited AML cell growth, including tumor volume and weight in vivo. In addition, the body weights of the tumor-bearing mice did not change significantly during the duration of study (Fig. 6c). The expression of Bcl-xl, Cyclin E, and PCNA with combined radotinib and Ara-C treatment was significantly decreased in tumor tissues isolated from the mice, as shown in Fig. 6d-e. Further, the expression of Bax and p21, as a well-known CDK inhibitor, was significantly increased in tumor tissues with combined treatment comprising radotinib and Ara-C. In addition, the expression of TUNEL-positive cells was significantly amplified, while the expression of PCNA-positive cells was dramatically reduced in the tumor tissue with combined treatment of radotinib and Ara-C (Fig. 6f-g). In particular, the combination of the two drugs was shown to lead to the profound inhibition of acute myeloid leukemia tumor growth (Fig. 6).

### Radotinib and Ara-C sensitize AML cells to chemotherapeutic agents including daunorubicin or idarubicin

Finally, we performed cell viability tests to determine the efficacy of a combination of radotinib and various anti-cancer drugs commonly used for AML therapy. Daunorubicin (DNR) is a chemotherapy medication used to treat cancer. It is specifically used for AML, acute lymphoblastic leukemia, CML [21]. Moreover, idarubicin (Idar) is an anthracycline antileukemic drug. It inserts itself into DNA and prevents DNA unwinding by interfering with the enzyme topoisomerase II [22]. It is also an analog of daunorubicin. As shown in Figs. 1, 2, 3, 4 and 5, radotinib enhanced sensitivity to the chemotherapeutic agent Ara-C in AML cells. Moreover, triple combination with radotinib, Ara-C, and DNR or Idar significantly inhibited the viability of AML cells (Supplementary Fig. 2). Therefore, these results indicate that a combination of radotinib and Ara-C can sensitize cells to chemotherapeutic agents such as DNR or Idar in AML cells, as well as HL60 and HEL92.1.7 cells.

## Discussion

Generally, apoptosis is a form of programmed cell death that occurs in multicellular organisms. Moreover, it is a key regulator of physiological growth control and the regulation of tissue homeostasis for the elimination of damaged, old, or infected cells [23, 24]. It is well known that activating the apoptosis pathway in various cancers can make cancer treatments successful, whereas inhibiting the activation of apoptosis leads to cancer resistance, which in turn makes cancer treatment difficult [23, 25]. Especially, targeting apoptosis proteins in hematologic malignancies has been shown to be very useful [24, 26].

The induction of apoptosis is a cell suicide mechanism and an ideal way to treat cancer including AML [27]. Basically, killing tumor cells with anticancer therapies commonly used for the treatment of cancer, such as chemotherapy, gamma-irradiation, and immunotherapy, among others, is mainly mediated by triggering apoptosis, wherein these modalities activate the cell’s intrinsic cell death process [28]. The activation of caspases is often impaired in human cancers and contributes to cancer formation, progression, and therapy resistance [27]. Therefore, understanding the molecular mechanisms that regulate caspase activation in cancer cells is very important. Thus, the modulation of caspase activation including apoptosis represents a promising approach for the development of new therapeutic opportunities to induce cancer cell death.

The combination of BCR-ABL inhibitors such as dasatinib and imatinib, with other used chemotherapeutic agents for AML is not new. According to Dos Santos et al., it has already been shown that the combination of dasatinib with daunorubicin or Ara-C also resulted in significantly increased AML CD34^+^ cell death [29]. And chemotherapy plus dasatinib provided excellent outcomes for both younger and older patients with or without KIT mutation [30]. Also, the Ara-C and imatinib showed synergistic effects in vitro [31]. According to a phase 1 study of imatinib mesylate in combination with cytarabine and daunorubicin, especially for c-kit positive recurrent acute myeloid leukemia, cytotoxic therapy that includes imatinib mesylate for relapsed AML was effective [32]. In addition, the combination of DNR and the novel BCR-ABL inhibitor ponatinib showed the AML cell death [33]. We have previously shown that radotinib significantly increases cytotoxicity or apoptosis in AML and CML cells [17]. Radotinib also acts as a CDK inhibitor, which strongly inhibits AML cell proliferation [17]. Alternatively, it functions as an AURKA inhibitor, which suppresses the expression of AURKA and related proteins including Bora, polo-like kinase 1, and TPX2 [34]. Moreover, radotinib induces apoptosis directly in cells differentiated from AML blasts [16]. In addition, we previously confirmed that c-KIT (CD117) could be targeted by radotinib, acting as a c-KIT inhibitor or HSP90 inhibitor in c-KIT-positive AML cells [18, 19]. Previous studies have shown that radotinib promotes AML cell death through various cellular mechanisms. However, little is known about the effects of the combination of radotinib and Ara-C on cell death and cell cycle distribution in AML cells.

AML is a heterogeneous group of rapidly growing cancers of myeloid progenitor cells [2, 35]. Ara-C has remained the backbone of chemotherapy for adult AML patients for decades [6]. Especially, high dose Ara-C is a part of an induction regimen as a first-line therapy for AML [36]. However, the resistance of AML cells to Ara-C chemotherapy is one of the most important reasons for relapse or chemo-refractoriness in AML patients [37]. For that reason, combination therapy has become the standard therapy for the treatment of several different cancers [38]. Therefore, it is of utmost importance to discover or develop drugs to maximize the effects of Ara-C. Clinicians, as well as scientists involved in basic research, interested in the treatment of AML, have focused on developing or discovering drugs that can be used in combination with Ara-C. Much attention has been given to the combination of two or more drugs, including Ara-C. Such examples include a combination of Ara-C and differentiating agents, a combination of Ara-C and DNA hypomethylating agents, and a combination of Ara-C and HSP90 inhibitors. Briefly, low-dose Ara-C combined with differentiating drugs or DNA hypomethylating agents comprises a potential regimen to treat AML patients who are unfit for high-intensity chemotherapy [39]. Phase I and pharmacological studies of Ara-C and tanespimycin, known as an HSP90 inhibitor, have been performed on relapsed and refractory acute leukemia [40, 41], and in particular, for patients with AML (except M3) or acute lymphocytic leukemia. Therefore, the combination of Ara-C and other drugs has been shown to be quite effective in treating acute leukemia. In this regard, our results pertaining to the combined use of Ara-C and radotinib suggest the successful development of a potential novel chemotherapeutic method for the treatment of AML. To our knowledge, this is the first discovery and study of this particular combination of drugs for AML therapy.

According to our results, radotinib enhanced Ara-C-induced AML cell death in diverse cell lines and BMCs from AML patients (Fig. 1). Moreover, combined treatment with radotinib and Ara-C had a synergistic effect on HL60 cell viability (combination index: 0.525, Supplementary Fig. 3). Further, radotinib and Ara-C significantly induced apoptosis in HL60 and HEL92.1.7 cells via the activation of the mitochondrial- and caspase-dependent apoptosis pathway (Figs. 2, 3 and 4). Moreover, combined treatment with both drugs exerted a synergistic effect on G_0_/G_1_ phase arrest of AML cells via the induction of p21 and p27, as shown in Fig. 5. Specifically, radotinib/Ara-C-induced bax expression was blocked by bongkrekic acid (Fig. 3e), and capase-3 activity was blocked by Z-VAD-FMK (Fig. 4f). Also we showed that Z-VAD-FMK and bongkrekic acid reversed the radotinib/Ara-C-induced effects including apoptotic proteins (cleaved PARP-1 and cleaved caspase-3; Fig. 4g and h), cell cycle markers (p21 and p27; Fig. 5d and e). These results showed mitochondrial-dependent apoptosis and G_0_/G_1_ phase cell cycle arrest were contributed to radotinib/Ara-C mediated anti-AML activity (Figs. 4 and 5). In addition, the suppressive effect of a combination of radotinib and Ara-C, on AML cell growth, was also demonstrated using in vivo xenograft models (Fig. 6). In addition, radotinib and Ara-C sensitized AML cells to daunorubicin- or idarubicin-induced cell death (Supplementary Fig. 2). It can, thus, be concluded that radotinib enhances Ara-C-induced AML cell death via mitochondrial-dependent apoptosis and that radotinib in combination with Ara-C possesses a strong anti-AML activity. Moreover, these results suggest that a clinical evaluation of radotinib with Ara-C-based regimens for AML patients is warranted.

Cytarabine (Ara-C) has been used as a majority therapy for AML for a long time [5, 8]. However, the development of resistance and high rates of relapse is a significant obstacle to the successful treatment of AML [5, 42]. In particular, we understand that the failure of treatment of AML is very closely related to the resistance to the Ara-C. Given our past diverse results, there are a lot of potential for radotinib to play a role in Ara-C-resistant AML cells. And we think research from this point of view is a great idea. Therefore, we are also in the process of starting a mechanism study of the Ara-C resistance. And we plan to actively conduct research on the mechanism study of the Ara-C resistance this in the future.

## Conclusions

Therefore, our results can be concluded that radotinib in combination with Ara-C possesses a strong anti-AML activity. These results warrant the clinical evaluation of radotinib with Ara-C-based regimens in AML patients.

## Supplementary Information

**Additional file 1: Supplementary Table 1.** Information of AML patients.

**Additional file 2: Supplementary Figure 1.** Combination of radotinib and Ara-C inhibits HL60 cell proliferation. Cells were stimulated with various concentrations of 0, 10, 30, 40 and 50 μM radotinib and 0, 40, 80, 120 and 160 nM Ara-C for 48 h. The cytotoxicity was then evaluated by a cell viability assay. (A) Dose-dependent responses of radotinib on cell viability. (B) Dose-dependent responses of Ara-C on cell viability. (C) Treatment of radotinib and/or Ara-C at 48 h. Representative data are shown for at least three independent experiments. These data represent the means ± SEM. Significantly different from the control (*) or combination of radotinib and Ara-C (#); *: *P*, 0.05; ***, ###: *P*, 0.001. C: DMSO-control, R: radotinib, A: Ara-C.

**Additional file 3: Supplementary Figure 2.** Radotinib and Ara-C sensitize the chemotherapeutic agents including daunorubicin (DNR) or idarubicin (Idar) in AML cells. (A) HL60 cells were cultured with 3 μM radotinib, 10 nM Ara-C and 50 nM DNR for 48 h. The cell viability was then evaluated by an MTS assay. Triple combination of radotinib, Ara-C and DNR on cell viability is more potent. (B) HEL92.1.7 cells were cultured with 5 μM radotinib, 10 nM Ara-C and 75 nM DNR for 48 h. Triple combination of radotinib, Ara-C and DNR on cell viability is more potent (C) HEL92.1.7 cells were cultured with 5 μM radotinib, 10 nM Ara-C and 2 nM idarubicin for 48 h. Triple combination of radotinib, Ara-C and idarubicin on cell viability is more powerful. These data represent the means ± SEM. Significantly different from control (*) or triple combination of radotinib, Ara-C and DNR/or idarubicin (#); ***, ###: *P* < 0.001.

**Additional file 4: Supplementary Figure 3.** Isobologram analysis of radotinib and Ara-C combination on AML cell death. Cell viability assay by radotinib and Ara-C was analyzed in HL60 cells. Cells were seeded (density, 2 × 10^4^ cells/well) in 96-well plates containing 200 μl medium per well and were incubated with diverse concentration of radotinib and/or Ara-C for 48 h at 37 °C. CellTiter 96 solution (20 μl; Promega, Madison, WI, USA) was added directly to each well, and the plates were incubated for 4 h in a humidified atmosphere of 5% CO_2_ at 37 °C. Absorbance was measured at 490 nm by using SpectraMax iD3 Microplate Reader (Molecular Devices, San Jose, CA, USA). We found the strong synergism on radotinib and Ara-C combination on AML cell death. Fifty % of inhibition concentration (IC_50_) on AML cell death in HL60 cells: Radotinib only, 200 μM; Ara-C only, 140 nM; combination of radotinib and Ara-C = 5 μM + 70 nM. Combination Index: 0.52).

**Additional file 5: Supplementary Figure 4.** Original western blots used for Fig. 3c, d and e. The blots were developed using the ChemiDoc™ Touch Imaging System, and analyzed with the Image Lab™ Software. The red boxes indicate the cropped regions used in the representative figures.

**Additional file 6: Supplementary Figure 5.** Original western blots used for Fig. 4c, g and h. The blots were developed using the ChemiDoc™ Touch Imaging System, and analyzed with the Image Lab™ Software. The red boxes indicate the cropped regions used in the representative figures.

**Additional file 7: Supplementary Figure 6.** Original western blots used for Fig. 5b, c, d and e. The blots were developed using the ChemiDoc™ Touch Imaging System, and analyzed with the Image Lab™ Software. The red boxes indicate the cropped regions used in the representative figures.

**Additional file 8: Supplementary Figure 7.** Original western blots used for Fig. 6d and e. The blots were developed using the ChemiDoc™ Touch Imaging System, and analyzed with the Image Lab™ Software. The red boxes indicate the cropped regions used in the representative figures.

**Additional file 9: Supplementary Table 2.** Supplementary Methods.

## Data Availability

The datasets used and/or analyzed during the current study are available from the corresponding author on reasonable request.

## Abbreviations

AML: Acute myeloid leukemia
CML-CP: Chronic phase-chronic myeloid leukemia (CML-CP)
BM: Bone marrow
BMCs: Bone marrow cells
PCNA: Proliferating cell nuclear antigen
DNR: Daunorubicin; Idar, idarubicin
