# Supplementary material for: Radotinib enhances cytarabine (Ara-C)-induced acute myeloid leukemia cell death
Source: BMC Cancer. 2020 Dec 4;20:1193. doi: 10.1186/s12885-020-07701-8 (PMC7718665; doi:10.1186/s12885-020-07701-8)
Supplement: Supplementary file 1 — Additional file 1: Supplementary Table 1. Information of AML patients. [file 12885_2020_7701_MOESM1_ESM.docx]

**Supplementary Table 1. Information of AML patients.**

| **UPN** | **Disease** | **Cell source** | **Blast (%)** | **Karyotype** |
| --- | --- | --- | --- | --- |
| **1** | **AML, minimal differentiation** | **BM** | **91.5** | **46,XY[20]** |
| **2** | **AML with maturation** | **BM** | **66** | **46,XY[30]** |
| **3** | **AML, minimal differentiation** | **BM** | **80** | **46,XX[7]** |
| **4** | **Acute myeloid leukemia without maturation** | **BM** | **73** | **46,XY[20]** |
| **5** | **AML with maturation** | **BM** | **82.5** | **46,XX[8]** |

AML, acute myeloid leukemia; BM, bone marrow.

**Supplementary Table 2.** Supplementary Methods.

| **Experiments** | **Number of mice** |
| --- | --- |
| *In vivo* test of Tumor cell growth | 12 mice total:  4 mice transplanted with HEL92.1.7 tumor cell with 1×10^7^  4 mice transplanted with HEL92.1.7 tumor cell with 1×10^7^  4 mice transplanted with HEL92.1.7 tumor cell with 3×10^7^ |
| *In vivo* experiment of radotinib dosage determination | 12 mice total:  3 mice treated with the vehicle (0.5% carboxymethylcellulose, orally)  9 mice treated with each of 30, 50 and 100 mg/kg radotinib, orally |
| *In vivo* experiment of Ara-C dosage determination | 12 mice total:  3 mice treated with the vehicle (DW, intraperitoneally)  9 mice treated with each of 50 and 100 mg/kg Ara-C, intraperitoneally |
| *In vivo* preclinical study of combination efficacy | 72 mice total:  18 mice treated with the vehicle (0.5% carboxymethylcellulose)  18 mice treated with radotinib only (50 mg/kg; orally)  18 mice treated with Ara-C (50 mg/kg; intraperitoneally)  18 mice treated with radotinib and Ara-C in combination |
