# Supplementary figures and images for: Radotinib enhances cytarabine (Ara-C)-induced acute myeloid leukemia cell death
Source: BMC Cancer. 2020 Dec 4;20:1193. doi: 10.1186/s12885-020-07701-8 (PMC7718665; doi:10.1186/s12885-020-07701-8)

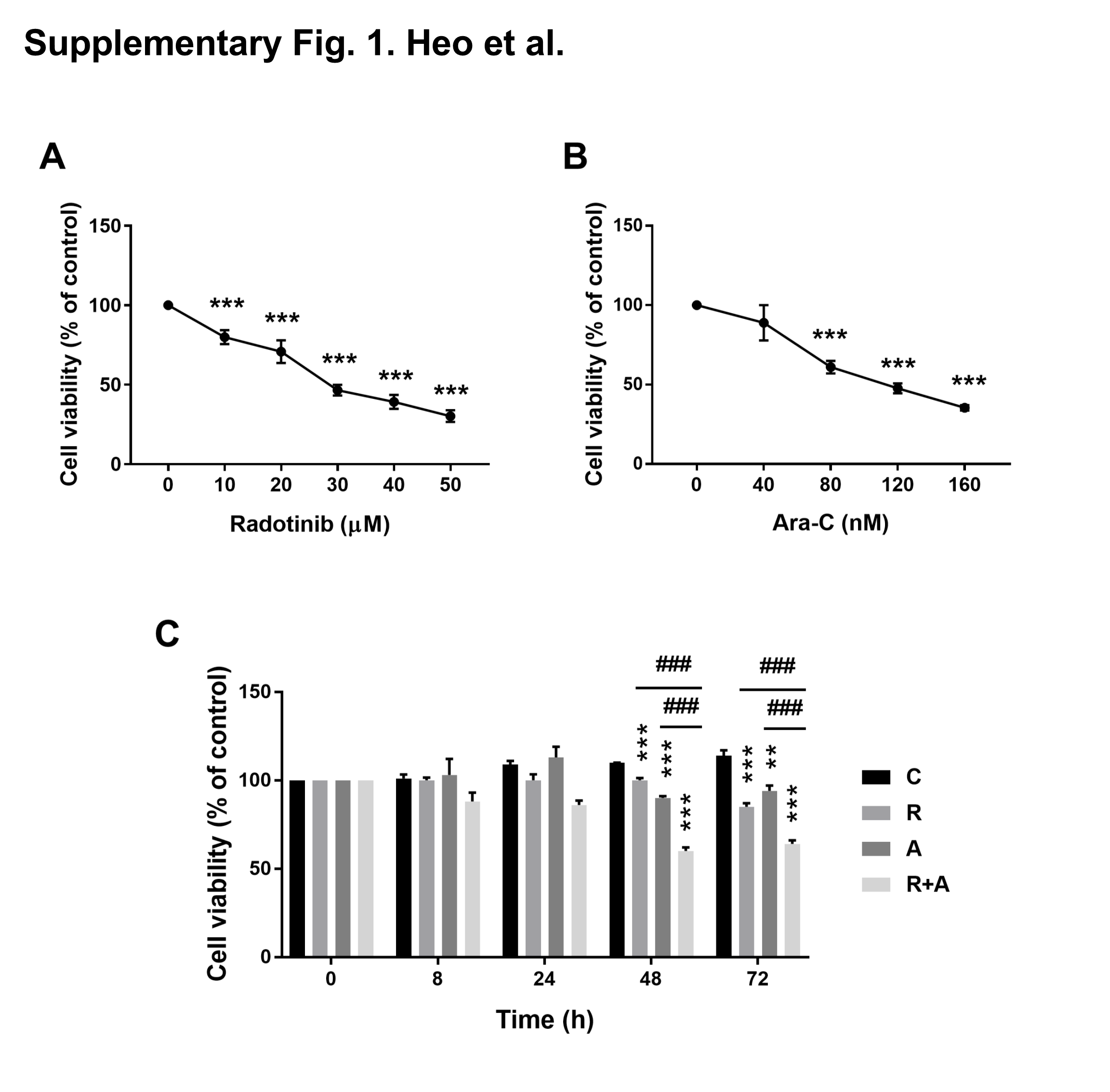

Supplement: Supplementary file 2 — Additional file 2: Supplementary Figure 1. Combination of radotinib and Ara-C inhibits HL60 cell proliferation. Cells were stimulated with various concentrations of 0, 10, 30, 40 and 50 μM radotinib and 0, 40, 80, 120 and 160 nM Ara-C for 48 h. The cytotoxicity was then evaluated by a cell viability assay. (A) Dose-dependent responses of radotinib on cell viability. (B) Dose-dependent responses of Ara-C on cell viability. (C) Treatment of radotinib and/or Ara-C at 48 h. Representative data are shown for at least three independent experiments. These data represent the means ± SEM. Significantly different from the control (*) or combination of radotinib and Ara-C (#); *: P, 0.05; ***, ###: P, 0.001. C: DMSO-control, R: radotinib, A: Ara-C. [file 12885_2020_7701_MOESM2_ESM.tif]

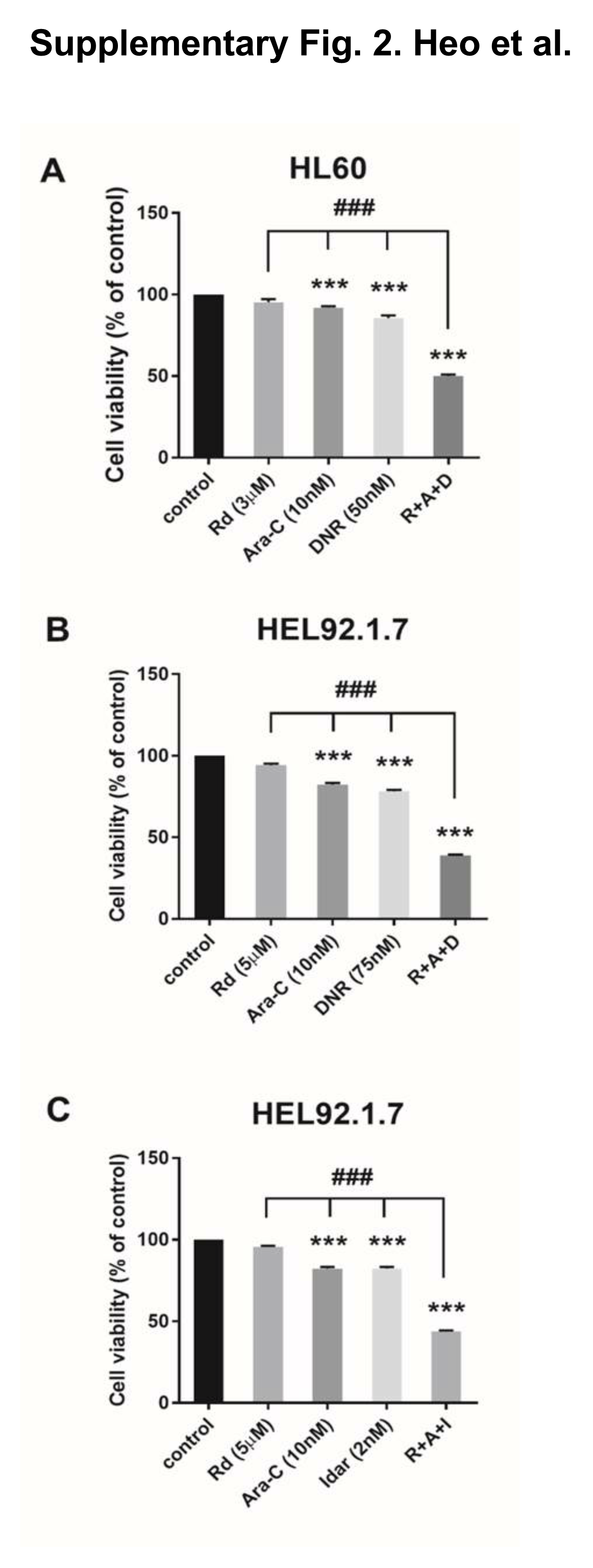

Supplement: Supplementary file 3 — Additional file 3: Supplementary Figure 2. Radotinib and Ara-C sensitize the chemotherapeutic agents including daunorubicin (DNR) or idarubicin (Idar) in AML cells. (A) HL60 cells were cultured with 3 μM radotinib, 10 nM Ara-C and 50 nM DNR for 48 h. The cell viability was then evaluated by an MTS assay. Triple combination of radotinib, Ara-C and DNR on cell viability is more potent. (B) HEL92.1.7 cells were cultured with 5 μM radotinib, 10 nM Ara-C and 75 nM DNR for 48 h. Triple combination of radotinib, Ara-C and DNR on cell viability is more potent (C) HEL92.1.7 cells were cultured with 5 μM radotinib, 10 nM Ara-C and 2 nM idarubicin for 48 h. Triple combination of radotinib, Ara-C and idarubicin on cell viability is more powerful. These data represent the means ± SEM. Significantly different from control (*) or triple combination of radotinib, Ara-C and DNR/or idarubicin (#); ***, ###: P < 0.001. [file 12885_2020_7701_MOESM3_ESM.tif]

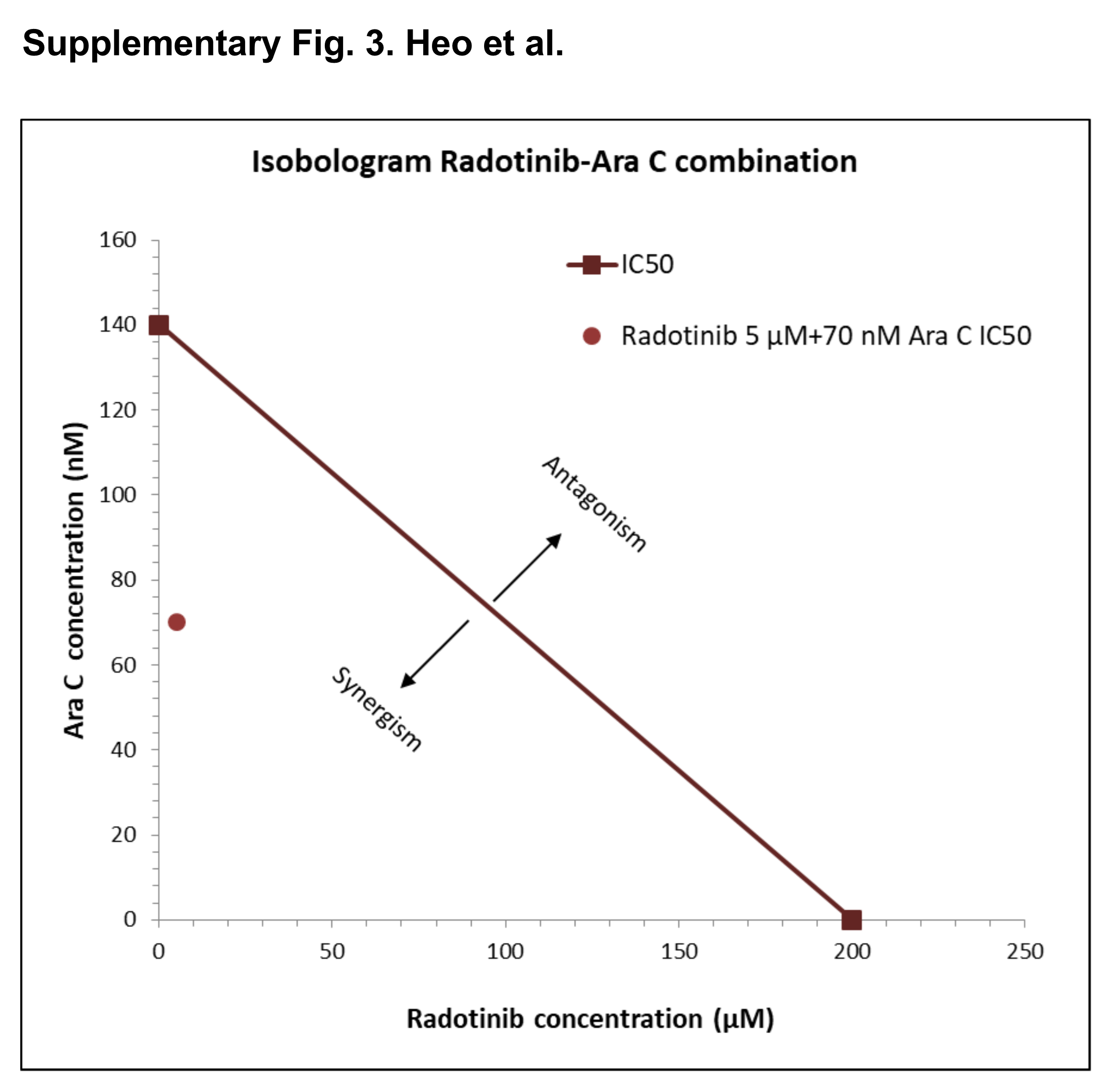

Supplement: Supplementary file 4 — Additional file 4: Supplementary Figure 3. Isobologram analysis of radotinib and Ara-C combination on AML cell death. Cell viability assay by radotinib and Ara-C was analyzed in HL60 cells. Cells were seeded (density, 2 × 10^4^ cells/well) in 96-well plates containing 200 μl medium per well and were incubated with diverse concentration of radotinib and/or Ara-C for 48 h at 37 °C. CellTiter 96 solution (20 μl; Promega, Madison, WI, USA) was added directly to each well, and the plates were incubated for 4 h in a humidified atmosphere of 5% CO2 at 37 °C. Absorbance was measured at 490 nm by using SpectraMax iD3 Microplate Reader (Molecular Devices, San Jose, CA, USA). We found the strong synergism on radotinib and Ara-C combination on AML cell death. Fifty % of inhibition concentration (IC50) on AML cell death in HL60 cells: Radotinib only, 200 μM; Ara-C only, 140 nM; combination of radotinib and Ara-C = 5 μM + 70 nM. Combination Index: 0.52). [file 12885_2020_7701_MOESM4_ESM.tif]

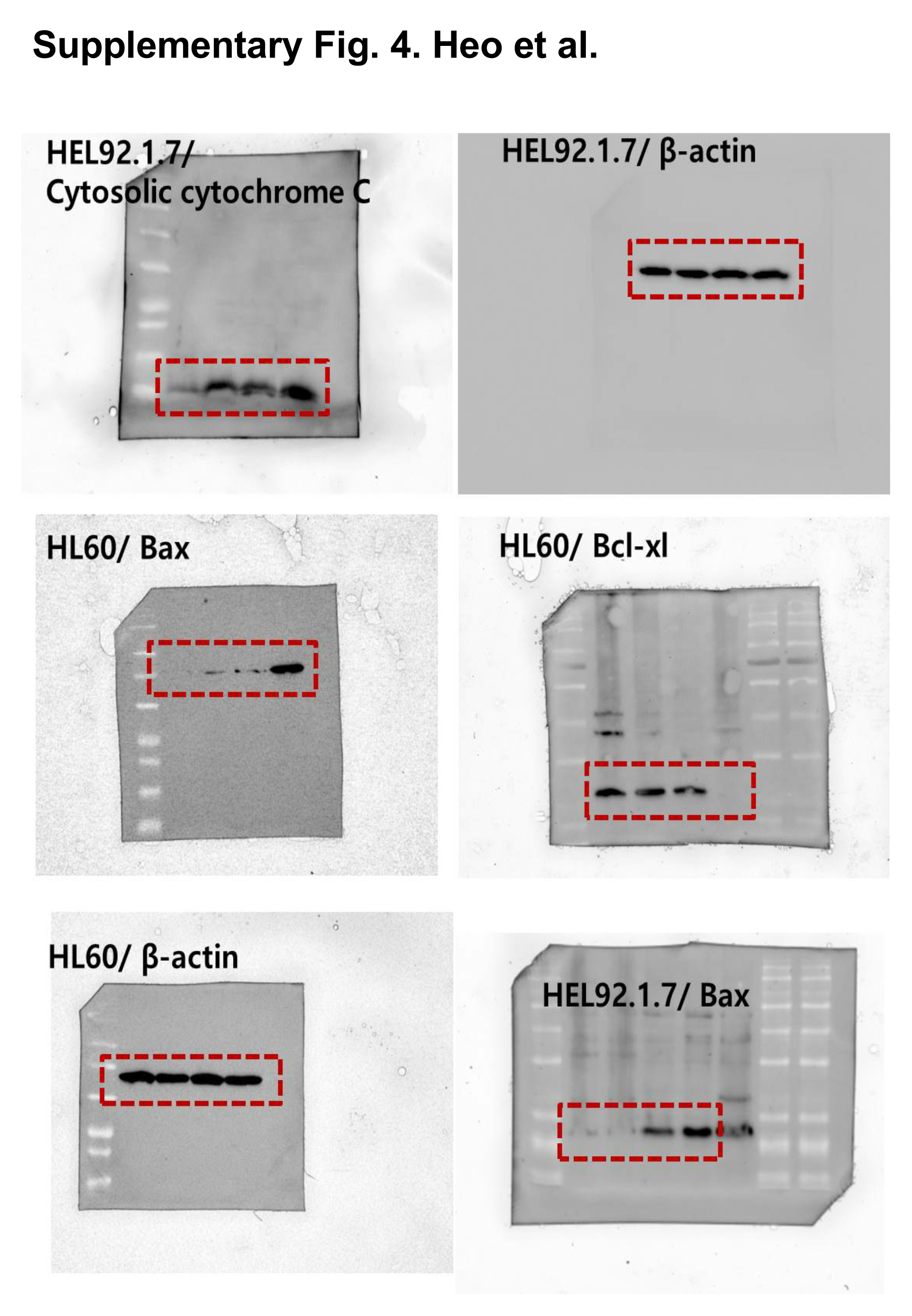

Supplement: Supplementary file 5 — Additional file 5: Supplementary Figure 4. Original western blots used for Fig. 3c, d and e. The blots were developed using the ChemiDoc™ Touch Imaging System, and analyzed with the Image Lab™ Software. The red boxes indicate the cropped regions used in the representative figures. [file 12885_2020_7701_MOESM5_ESM.zip › Supplementary Fig 4-1R2.tif]

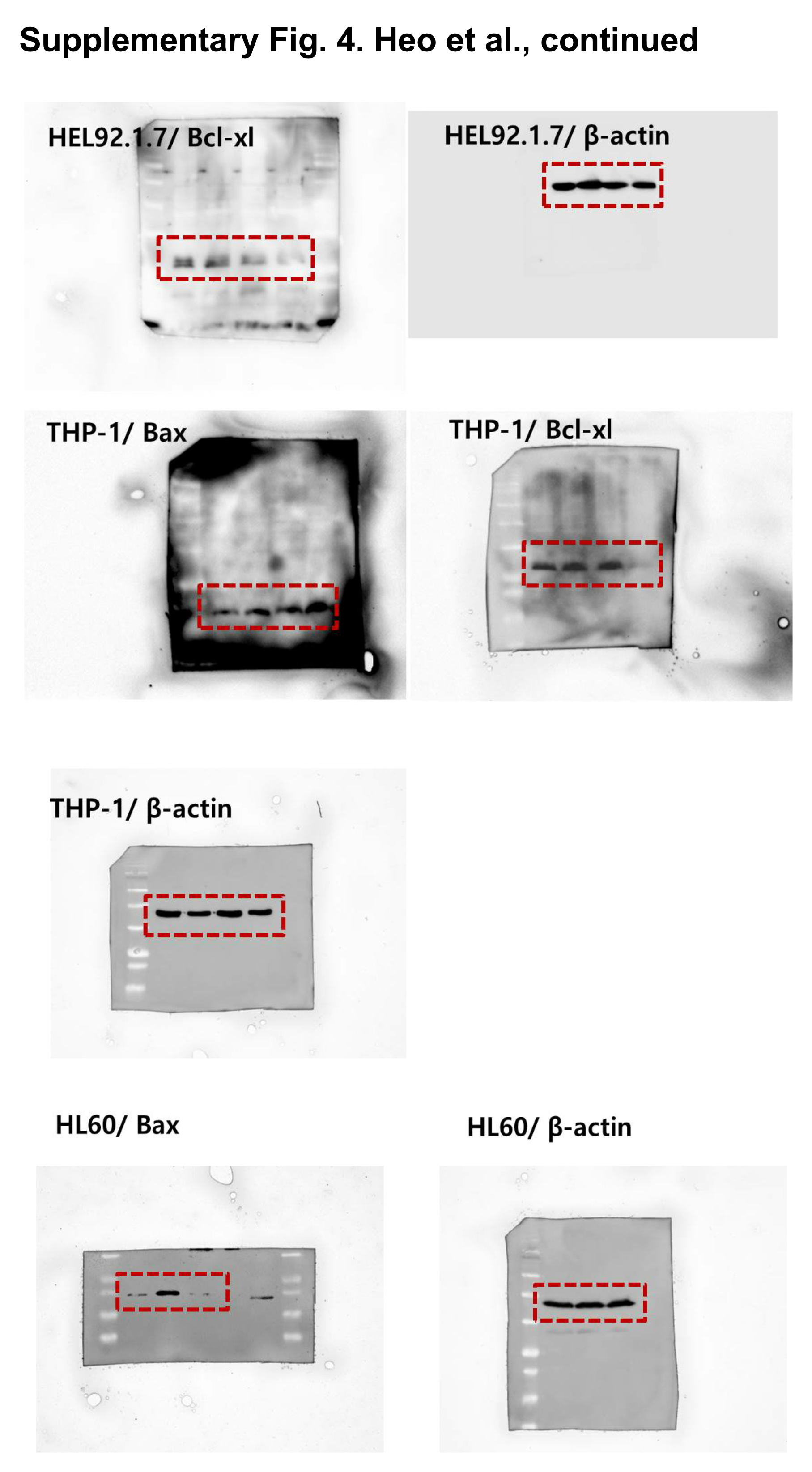

Supplement: Supplementary file 5 — Additional file 5: Supplementary Figure 4. Original western blots used for Fig. 3c, d and e. The blots were developed using the ChemiDoc™ Touch Imaging System, and analyzed with the Image Lab™ Software. The red boxes indicate the cropped regions used in the representative figures. [file 12885_2020_7701_MOESM5_ESM.zip › Supplementary Fig 4-2R2.tif]

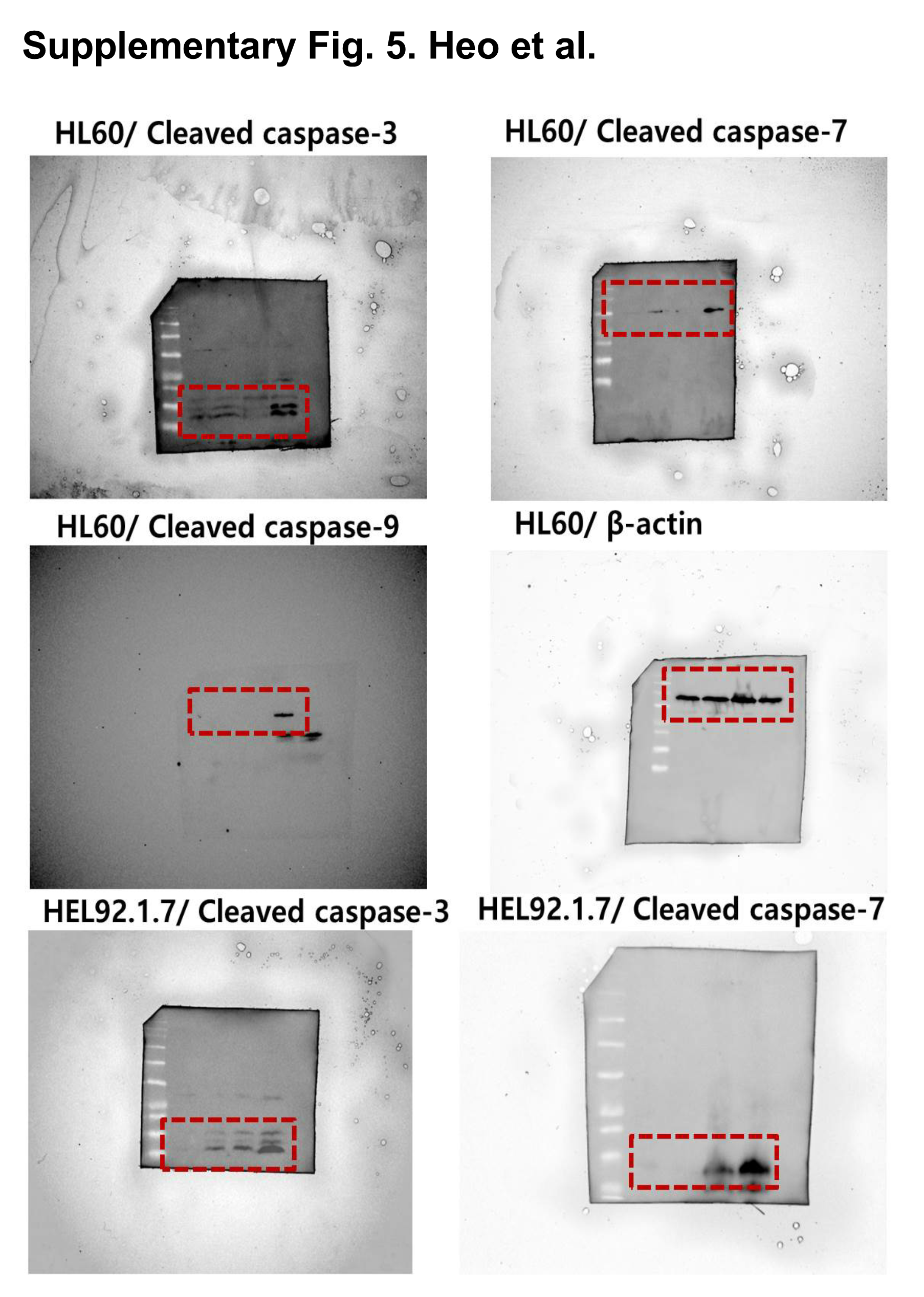

Supplement: Supplementary file 6 — Additional file 6: Supplementary Figure 5. Original western blots used for Fig. 4c, g and h. The blots were developed using the ChemiDoc™ Touch Imaging System, and analyzed with the Image Lab™ Software. The red boxes indicate the cropped regions used in the representative figures. [file 12885_2020_7701_MOESM6_ESM.zip › Supplementary Fig 5-1R2.tif]

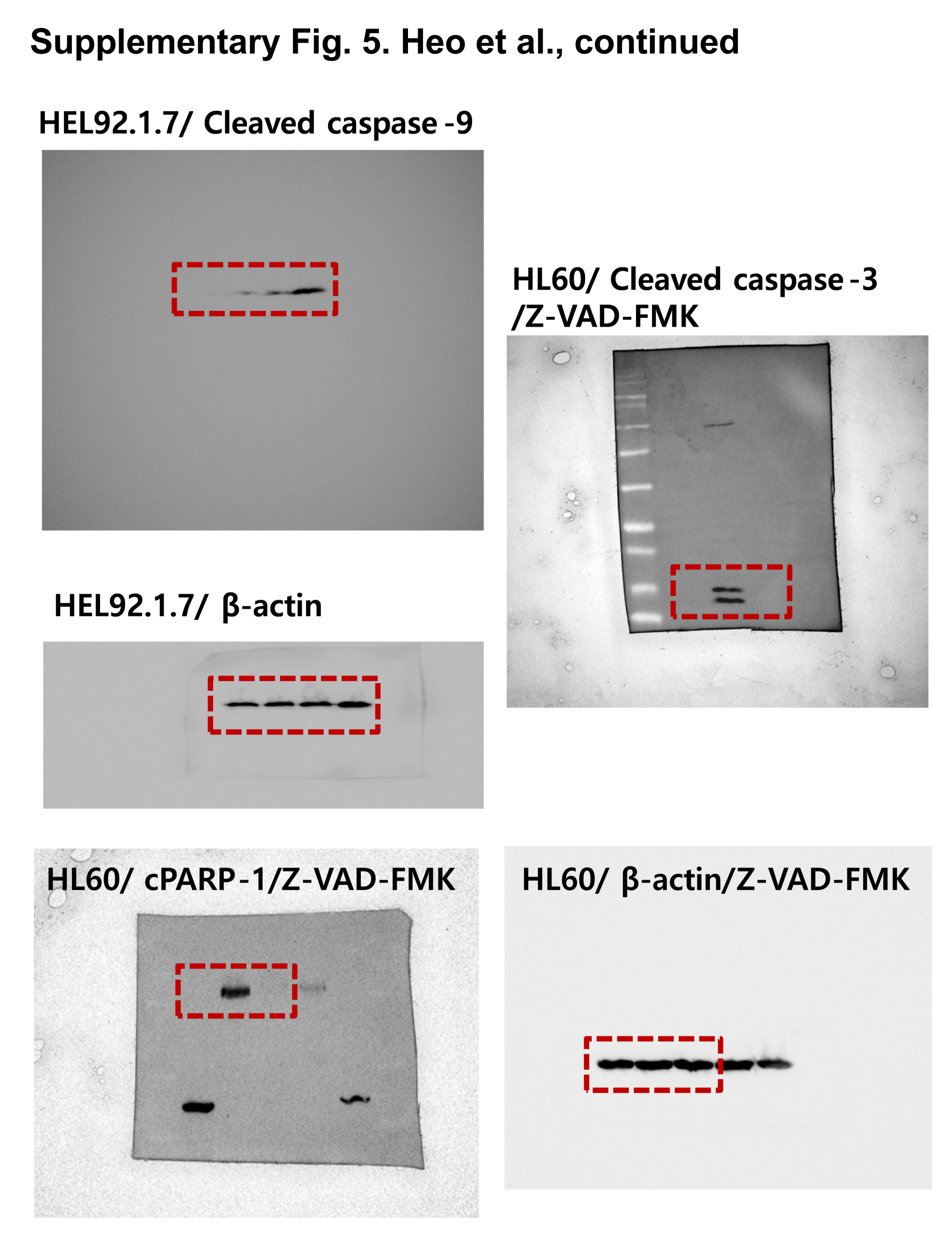

Supplement: Supplementary file 6 — Additional file 6: Supplementary Figure 5. Original western blots used for Fig. 4c, g and h. The blots were developed using the ChemiDoc™ Touch Imaging System, and analyzed with the Image Lab™ Software. The red boxes indicate the cropped regions used in the representative figures. [file 12885_2020_7701_MOESM6_ESM.zip › Supplementary Fig 5-2RR2.tif]

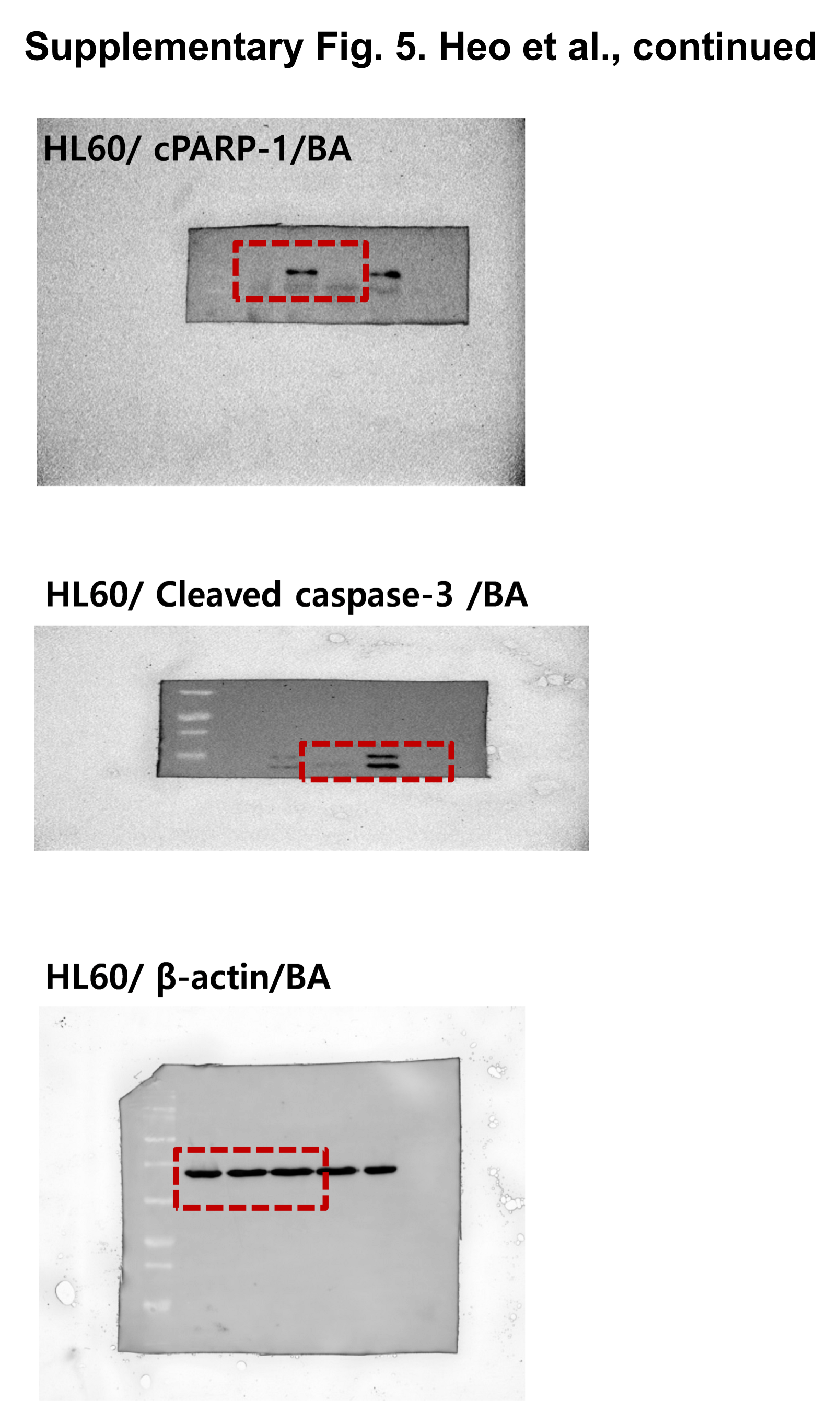

Supplement: Supplementary file 6 — Additional file 6: Supplementary Figure 5. Original western blots used for Fig. 4c, g and h. The blots were developed using the ChemiDoc™ Touch Imaging System, and analyzed with the Image Lab™ Software. The red boxes indicate the cropped regions used in the representative figures. [file 12885_2020_7701_MOESM6_ESM.zip › Supplementary Fig 5-3RR2.tif]

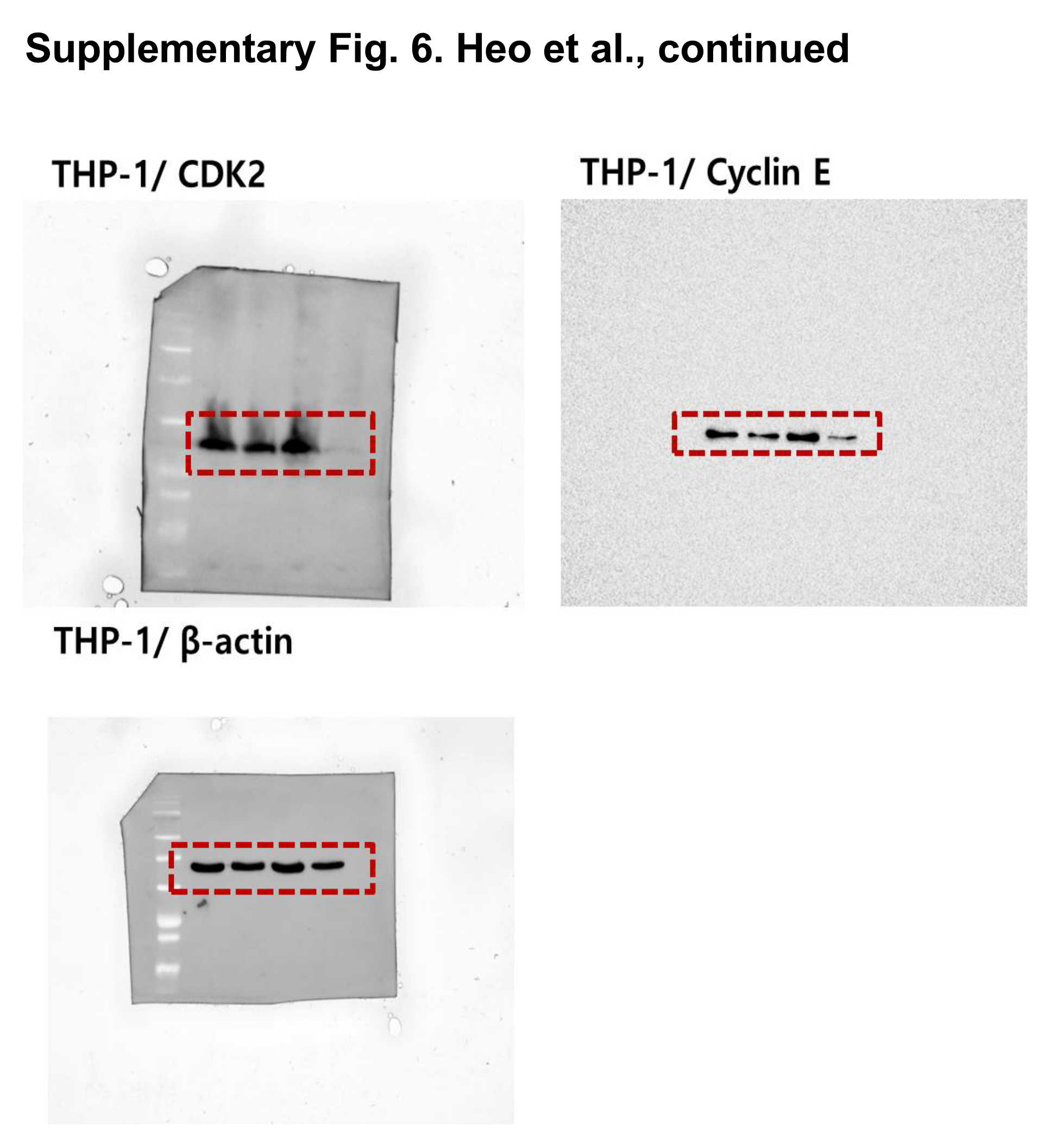

Supplement: Supplementary file 7 — Additional file 7: Supplementary Figure 6. Original western blots used for Fig. 5b, c, d and e. The blots were developed using the ChemiDoc™ Touch Imaging System, and analyzed with the Image Lab™ Software. The red boxes indicate the cropped regions used in the representative figures. [file 12885_2020_7701_MOESM7_ESM.zip › Supplementary Fig 6-2R2.tif]

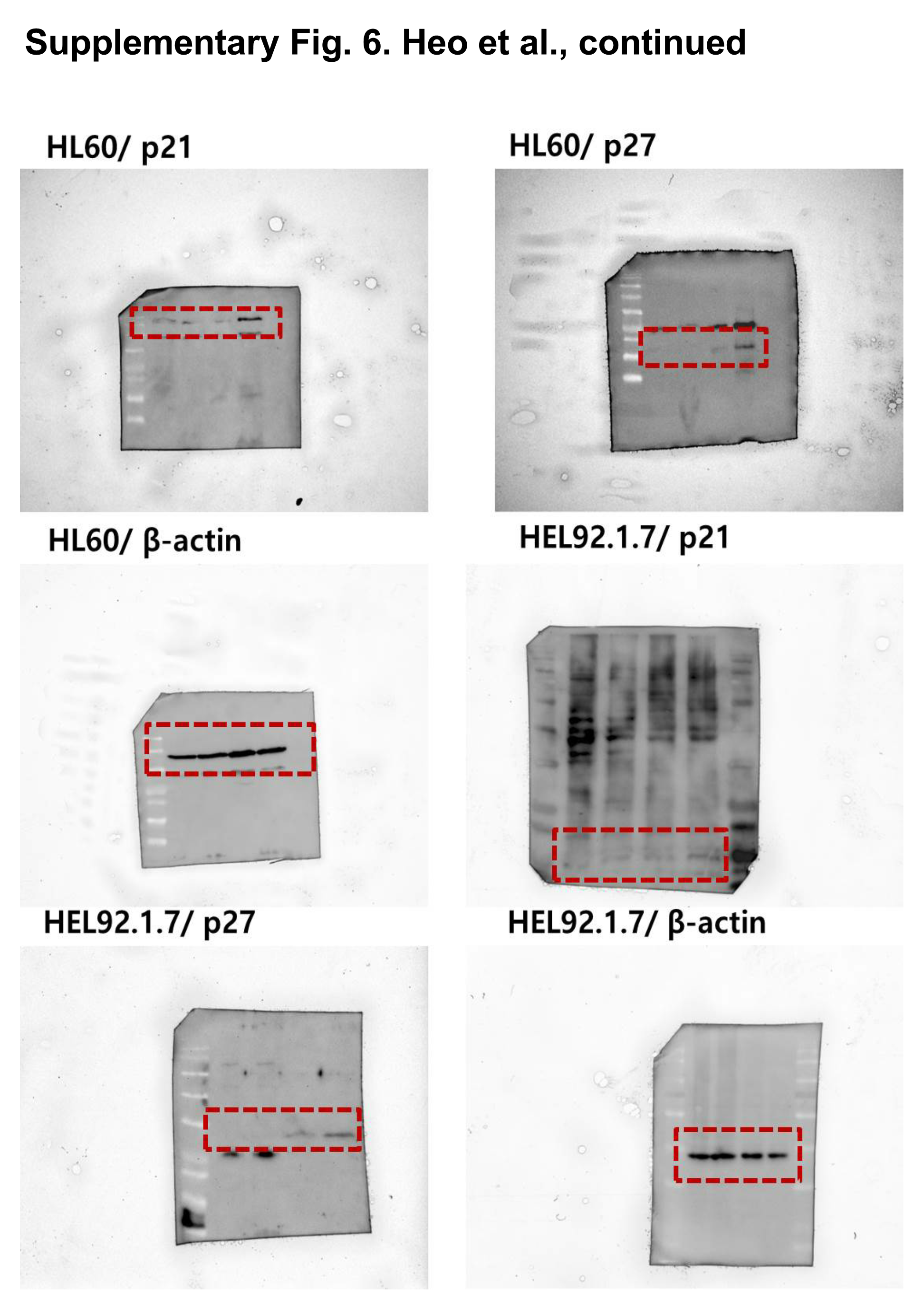

Supplement: Supplementary file 7 — Additional file 7: Supplementary Figure 6. Original western blots used for Fig. 5b, c, d and e. The blots were developed using the ChemiDoc™ Touch Imaging System, and analyzed with the Image Lab™ Software. The red boxes indicate the cropped regions used in the representative figures. [file 12885_2020_7701_MOESM7_ESM.zip › Supplementary Fig 6-3R2.tif]

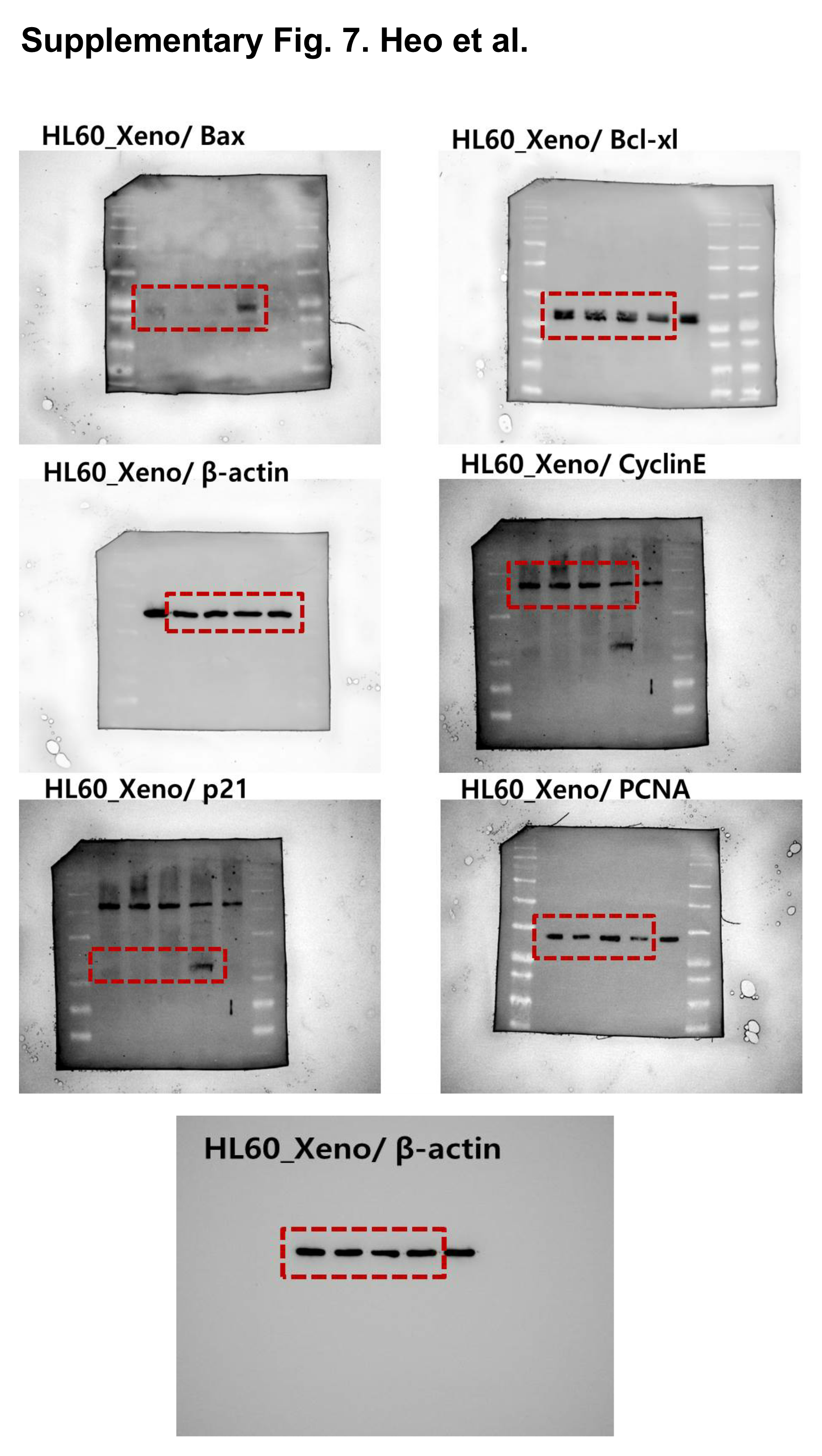

Supplement: Supplementary file 7 — Additional file 7: Supplementary Figure 6. Original western blots used for Fig. 5b, c, d and e. The blots were developed using the ChemiDoc™ Touch Imaging System, and analyzed with the Image Lab™ Software. The red boxes indicate the cropped regions used in the representative figures. [file 12885_2020_7701_MOESM7_ESM.zip › Supplementary Fig 7R2.tif]

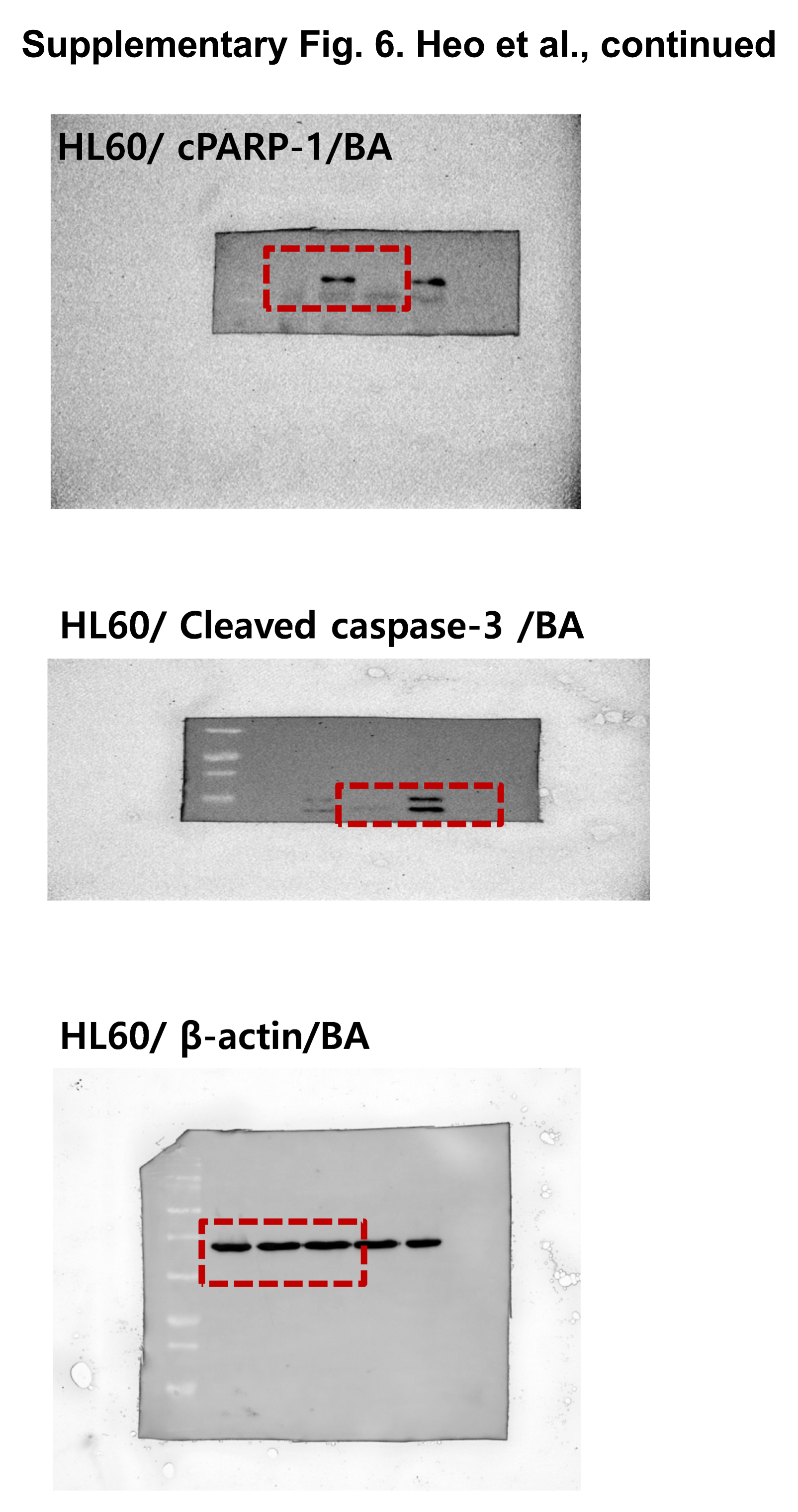

Supplement: Supplementary file 7 — Additional file 7: Supplementary Figure 6. Original western blots used for Fig. 5b, c, d and e. The blots were developed using the ChemiDoc™ Touch Imaging System, and analyzed with the Image Lab™ Software. The red boxes indicate the cropped regions used in the representative figures. [file 12885_2020_7701_MOESM7_ESM.zip › Supplementary Fig 6-4RR2.tif]
